# Dynamic Transcription Machineries Guide the Synthesis of Temporally Operating DNAzymes, Gated and Cascaded DNAzyme Catalysis

**DOI:** 10.1021/acsnano.2c10108

**Published:** 2022-12-28

**Authors:** Jiantong Dong, Itamar Willner

**Affiliations:** Institute of Chemistry, Center for Nanoscience and Nanotechnology, The Hebrew University of Jerusalem, Jerusalem 91904, Israel

**Keywords:** out-of-equilibrium, systems chemistry, network, RNase, kinetic simulation, RNA, DNA nanotechnology

## Abstract

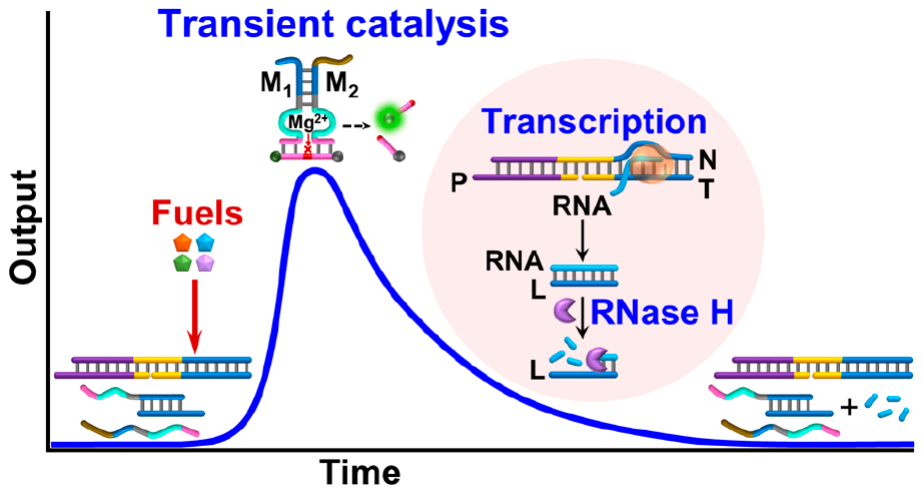

Transient transcription machineries play important roles in the dynamic modulation of gene expression and the sequestered regulation of cellular networks. The present study emulates such processes by designing artificial reaction modules consisting of transcription machineries that guide the transient synthesis of catalytic DNAzymes, the transient operation of gated DNAzymes, and the temporal activation of an intercommunicated DNAzyme cascade. The reaction modules rely on functional constituents that lead to the triggered activation of transcription machineries in the presence of the nucleoside triphosphates oligonucleotide fuel, yielding the transient formation and dissipative depletion of the intermediate DNAzyme(s) products. The kinetics of the transient DNAzyme networks are computationally simulated, allowing to predict and experimentally validate the performance of the systems under different auxiliary conditions. The study advances the field of systems chemistry by introducing transcription machinery-based networks for the dynamic control over transient catalysis—a primary step toward life-like cellular assemblies.

## Introduction

Transient transcription machineries play important roles in the dynamic modulation of gene expression and sequestered regulation of cellular processes.^1−4^ Spatial and temporal misregulation of gene expression programs leads to diverse diseases, and developing means to inhibit the misregulated transcription pathways is a scientific challenge.^5^ Indeed, mimicking transient dynamic machineries by artificial means and the development of methods to block and control gene expression circuitries is a key goal of systems chemistry.^6,7^ In addition, dynamic gene expression machineries play important roles in intercommunicating complex genetic networks and the fan-out or branched production of multifunctional proteins.^8−10^ Accordingly, modulating such processes by synthetic means provides primary steps toward synthetic cell functions—protocells.^11,12^ Recent advances in DNA nanotechnology used the information encoded in the base-sequence of the DNA biopolymer to assemble dynamic DNA circuitries and networks.^13−15^ The dynamic features of these systems were guided by the structural reconfiguration of single-stranded nucleic acid or duplex DNA scaffolds. Different triggers were used to reconfigure DNA structures including strand displacement,^16,17^ formation and dissociation of G-quadruplexes^18,19^ or triplex structures,^20^ and the use of light and photoisomerizable intercalators to stabilize/destabilize nucleic acid duplexes.^21^ These triggered DNA reconfiguration motives were applied for the dynamic assembly of complex reaction circuitries and programmed network assemblies.^22,23^ Dynamically triggered constitutional dynamic networks, revealing adaptive,^15,24^ hierarchically adaptive,^25^ feedback-driven,^26^ intercommunicated features,^27^ were reported and their use for programmed dynamic catalysis^28^ were demonstrated. Particularly, transient out-of-equilibrium, dissipative nucleic-acid-based networks attracted substantial recent research efforts.^29−32^ Enzyme-guided transient networks driven by ligase, endonucleases, or nickases were reported,^33−35^ and dynamic reaction circuits revealing oscillatory behaviors,^36,37^ gated and cascaded transient operations^38−40^ using dissipative reconfiguration of dynamic networks were achieved. Also, network-guided transient biocatalytic reaction modules dictating transient enzyme cascades,^41^ light-induced formation and dissipative depletion of microscale structures, e.g., microtubules,^42,43^ or nanoparticle aggregates,^44^ and transient enzyme-guided release and uptake of loads were demonstrated.^45,46^

The modulation of transcription machineries in nature by auxiliary triggers such as miRNA, plays important roles in gene expression.^47^ The modularity of transcriptional circuits provides a “toolbox” of nucleic acid structures for the catalytic synthesis of dynamically modulated transcriptional frameworks and artificial circuits.^48−51^ Indeed, transcriptional oscillators,^37,50^ transcriptional switches,^52^ and bistable gene-regulatory networks^53^ were reported. Nonetheless, while recent efforts addressed the dynamic control over transcriptional circuits and transcription/translation networks using fuel/antifuel strand displacement triggers,^54^ enzyme-driven assembly/disassembly of DNA-RNA nanotubes^55,56^ or colloidal nanoparticles,^57^ and the autoinhibited transcription of RNA in the presence of RNA polymerase aptamer as inhibitor,^58^ the transient, triggered activation of temporally operating catalytic agents and chemical transformations driven by transcriptional machineries is a challenging topic that needs to be addressed.

In the present study, we introduce a reaction module in which the nucleoside triphosphates (NTPs) fueled, triggered activation of a transcription machinery leads to the transient temporal formation of Mg^2+^-ion-dependent DNAzyme catalysts. The integration of RNase H in the system, which specifically cleaves RNA hybridized to DNA, leads to the dissipative depletion of the DNAzyme catalyst at the expense of “waste” products generated by the degradation of the transcribed RNA. By treatment of synthetically designed reaction modules with appropriate blockers, gated selective temporal operation of one of two dictated DNAzymes is demonstrated, and by coupling of two transcription machineries, the temporal cascaded transient operation of two dissipative DNAzymes is introduced. Previous reports discussed the application of DNAzymes as functional constituents to operate the transient reaction modules.^39,40^ Also, the transient synthesis of DNAzyme products by applying nucleic acid reaction modules, fuel-strands and nickase,^38^ or ATP-driven ligation and nickase^35^ as operators guiding the transient behavior of the reaction modules were demonstrated. The integration of the transcription machinery as a functional vehicle that drives the transient operation of DNAzyme would, however, advance the plethora of transcription-stimulated processes mimicking natural processes. The present study introduces the application of transcription machinery coupled to RNase as a mechanistic cycle for the transient operation of DNAzymes. Particularly, the transcription machinery/RNase system allows us to develop transient operating gated and cascaded DNAzyme networks as a primary model step toward life-like cellular transformations. The different dynamic reaction modules involving the transcription machinery-guided temporal synthesis and separation of the DNAzymes are accompanied by kinetic models that account for the kinetic behaviors of the transient networks. Computational simulations of the experimental results by the kinetic models not only provide a means to evaluate the rate constants of the subreactions involved in the dynamic operation of the systems but also allow to predict and experimentally validate the kinetic behaviors of the systems under different auxiliary conditions.

## Results and Discussion

### A Nucleotide Fuel Mixture Activates the Transcription Machinery to Guide the Synthesis of a Transiently Operating DNAzyme

**Figure 1:**
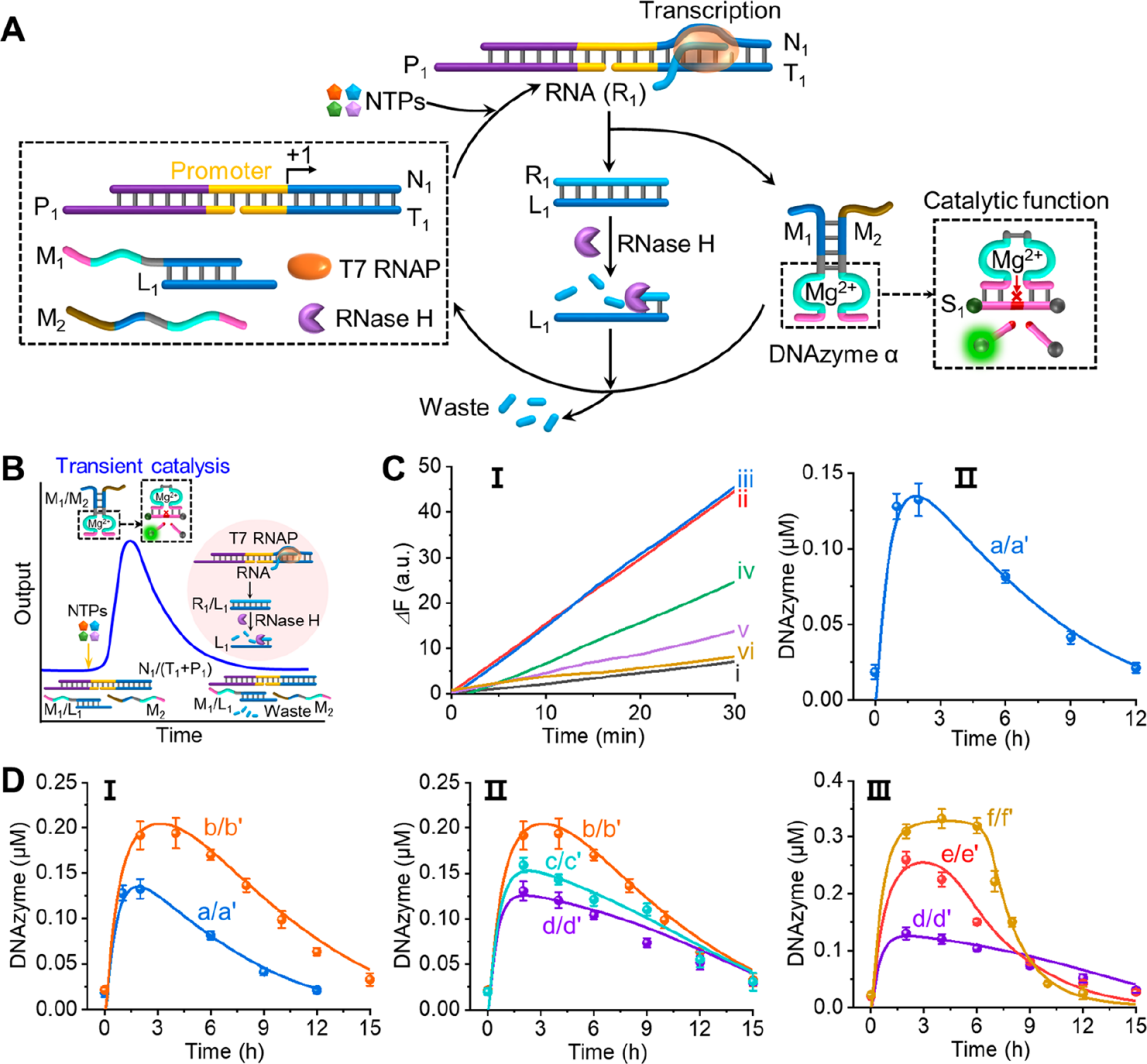
(A) Schematic reaction module driving the transient transcription machinery, leading to the dynamic formation and depletion of the Mg^2+^-ion-dependent DNAzyme (DNAzyme α). (B) Schematic reaction profile corresponding to the dissipative formation and depletion of an intermediate Mg^2+^-ion-dependent DNAzyme. (C) Panel I: Temporal, time-dependent fluorescence changes generated by the intermediate DNAzyme α, upon catalyzed cleavage of its fluorophore/quencher-modified substrate S_1_, formed at different time intervals of transient generation and depletion according to (A): (i) at *t* = 0, (ii) 1 h, (iii) 2 h, (iv) 6 h, (v) 9 h, and (vi) 12 h. Panel II: Temporal concentrations of DNAzyme α upon the transient operation of the reaction module in the presence of N_1_/T_1_+P_1_ = 0.2 μM, M_1_/L_1_ = 0.5 μM, M_2_ = 0.5 μM, NTPs = 0.5 mM, T7 RNAP = 2 U/μL (0.032 μM), and RNase H = 6 U/mL (0.199 nM). Transient dots, experimental data (error bars represent standard deviations of three measurements); solid transient curve, computationally simulated results using the kinetic model formulated on Figure S5. (D) Temporal concentrations corresponding to the intermediate transient DNAzyme α upon operating the reaction module shown in (A) under different auxiliary conditions. Dots (denoted by *x*) in different panels correspond to experimental data, and solid curves (denoted by *x*′) correspond to computationally simulated data. Panel I: a/a′, experimental and computational conditions as presented in (C); b′, computationally simulated data using the set of reaction rates derived for curve a′ but using NTPs = 1 mM; b, validated experimental results in the presence of NTPs = 1 mM. All other conditions are as described in (C). Panel II: b/b′ as stated in panel I; c′, computationally simulated data using the set of reaction rates derived for curve a′, c, validated experimental results, in the presence of NTPs = 1 mM, RNase H = 7 U/mL (0.233 nM); d′, computationally simulated data, d, validated experimental results, in the presence of NTPs = 1 mM, RNase H = 8 U/mL (0.266 nM). All other conditions are as described in (C). Panel III: d/d′ as stated in panel II; e′, computationally simulated data using the set of reaction rates derived for curve a′, e, validated experimental results, in the presence of NTPs = 1 mM, T7 RNAP = 3 U/μL (0.048 μM), RNase H = 8 U/mL (0.266 nM); f′, computationally simulated data, f, validated experimental results, in the presence of NTPs = 1 mM, T7 RNAP = 4 U/μL (0.064 μM), RNase H = 8 U/mL (0.266 nM). All other conditions are the same as those stated in (C). Error bars represent standard deviations of three measurements.

The transient synthesis of a Mg^2+^-ion-dependent DNAzyme by a transcription machinery is schematically outlined in Figure 1A. The reaction module consists of a transcription template consisting of a promoter strand N_1_ hybridized with two strands P_1_ and T_1_, a functional duplex composed of M_1_/L_1_, where M_1_ corresponds to a subunit of the Mg^2+^-ion-dependent DNAzyme α, a single strand M_2_ that corresponds to the second subunit of the DNAzyme, T7 RNA polymerase (RNAP), and RNase H. Within this configuration, caging M_1_ in the duplex structure M_1_/L_1_ prohibits the assembly of the DNAzyme structure. Subjecting the reaction module to the NTPs (as fuel) activates the transcription machinery that synthesizes RNA (R_1_). The resulting transcribed R_1_ is engineered to displace the duplex M_1_/L_1_ to yield R_1_/L_1_ and to release strand M_1_ that self-assembles into the M_1_/M_2_ DNAzyme structure. The resulting duplex R_1_/L_1_ is cleaved, however, by RNase H to release L_1_ that separates the intermediate Mg^2+^-ion-dependent DNAzyme, leading to the regeneration of the rest reaction module of the system. The activation of the transcription machinery in the reaction module with the NTPs fuel leads to the R_1_-stimulated transient synthesis of the Mg^2+^-ion-dependent DNAzyme intermediate that is dissipatively depleted by RNase H to regenerate the rest reaction module. By extruding samples from the dynamic reaction system, the transient formation and depletion of the Mg^2+^-ion-dependent DNAzyme α can be quantitatively probed by following the kinetics of the DNAzyme-stimulated cleavage of the fluorophore/quencher-modified substrate S_1_.

Figure 1B schematically depicts the transient kinetic formation and dissipative depletion of the intermediate DNAzyme driven by the NTPs-fueled transcription machinery and the accompanying RNase “waste” generating process that degrades the intermediate DNAzyme. Figure 1C, panel I, depicts the time-dependent fluorescence changes generated by the DNAzyme samples, withdrawn from the dynamic transcription machinery-triggered reaction module, where the time-dependent fluorescence changes correspond to cleavage of the substrate S_1_ by the DNAzyme α and represent the catalytic activity (concentration) of the DNAzyme in the samples. Evidently, the rates of time-dependent fluorescence changes increase for a time interval of 2 h and decay to the original value within a time interval of ca. 10 h. Using an appropriate calibration curve relating to the catalytic activities of the DNAzyme to the concentrations of the DNAzyme (Figure S1A), the temporal transient concentrations of the DNAzyme α upon formation and dissipative depletion were evaluated (Figure 1C, panel II, curve a). In a control experiment, Figure S2A, no synthesized DNAzyme activities were observed in the absence of added NTPs during the time interval of the experiment, confirming that the addition of NTPs triggers the transient generation of the DNAzyme. Furthermore, upon exhausting the NTPs in the first transient cycle, the temporal generation of the DNAzyme can be reactivated by the addition of the NTPs fuel (Figure S2B). Furthermore, it should be noted that RNase H does not affect the DNAzyme substrate^59^ (see Figure S3). (Also, for additional optimization of the system shown in Figure 1, the temporal reaction module was probed at additional auxiliary conditions displayed in Figure S4.) To account for the experimental transient behavior of the formation and dissipative depletion of the DNAzyme, we formulated a kinetic model that includes the set of subreactions participating in the transient process (Figure S5), and the experimental results were computationally simulated following the kinetic model. The computationally simulated transient behavior of the DNAzyme is displayed in Figure 1C, curve a′, overlaid on the experimental results. (In order to simulate the curves, a set of background experiments elucidating rate constants related to the kinetics and aimed to guide the effective simulation process were essential. This set of experiments and their significance were addressed in Figures S6–S11 and the accompanying discussions explaining these experiments, Supporting Information, pages S9–S18.) The set of computationally derived rate constants of the subreactions comprising the kinetic model are summarized in Table S1. (The sets of computationally derived rate constants of the background experiments are summarized in Tables S2 and S3). The kinetic model and the derived computationally simulated rate constants are of scientific value only if they have a predictive power on the kinetic behavior of the system under different auxiliary conditions that can be, subsequently, validated experimentally. Accordingly, we applied different auxiliary conditions to the transient appearance and depletion of the intermediate DNAzyme and probed the predicted transient behavior of the DNAzyme by applying the kinetic model and the derived set of simulated rate constants. We then validated the predicted kinetic behavior by experiments. The temporal concentrations of the DNAzyme were evaluated at the new auxiliary conditions (Figure S12). Figure 1D, panel I, solid line, depicts the computationally predicted transient behavior of the DNAzyme at the NTPs concentration of 1 mM (the set of rate constants were derived for NTPs concentration corresponding to 0.5 mM, curve a′). The dotted curve b depicts the experimentally validated results. Very good agreement between the predicted and experimentally validated results is observed. Figure 1D, panel II depicts, in curve b/b′, the computational and experimental results of the system at NTPs concentration of 1.0 mM and RNase H, 6 U/mL. The set of derived rate constants was applied to predict the transient behavior of the system at a RNase H concentration corresponding to 7 U/mL and 8 U/mL, respectively. The predicted transient results are displayed in panel II, curves c′ and d′. The predicted transient behaviors at these conditions were, then, validated and presented in curves c and d (dotted curves). As before, the experimental results overlay nicely the predicted computational results. Figure 1D, panel III, shows the computationally predicted transient behavior of the DNAzyme using the set of derived rate constants in the presence of T7 RNA polymerase, 2 U/μL, curve d′, 3 U/μL, curve e′, and 4 U/μL, curve f′, respectively, and the dotted curves correspond to the experimentally validated results, curves d, e, and f, respectively. The formulated kinetic model and the derived computationally simulated rate constants provide a solid computational framework to quantitatively predict the transient behavior of the system under different auxiliary conditions. (For further discussion of the kinetic models and simulations, see Supporting Information, pages S9–S18.)

### Gated Operation of Dissipative DNAzymes Guided by a Transcription Machinery

**Figure 2:**
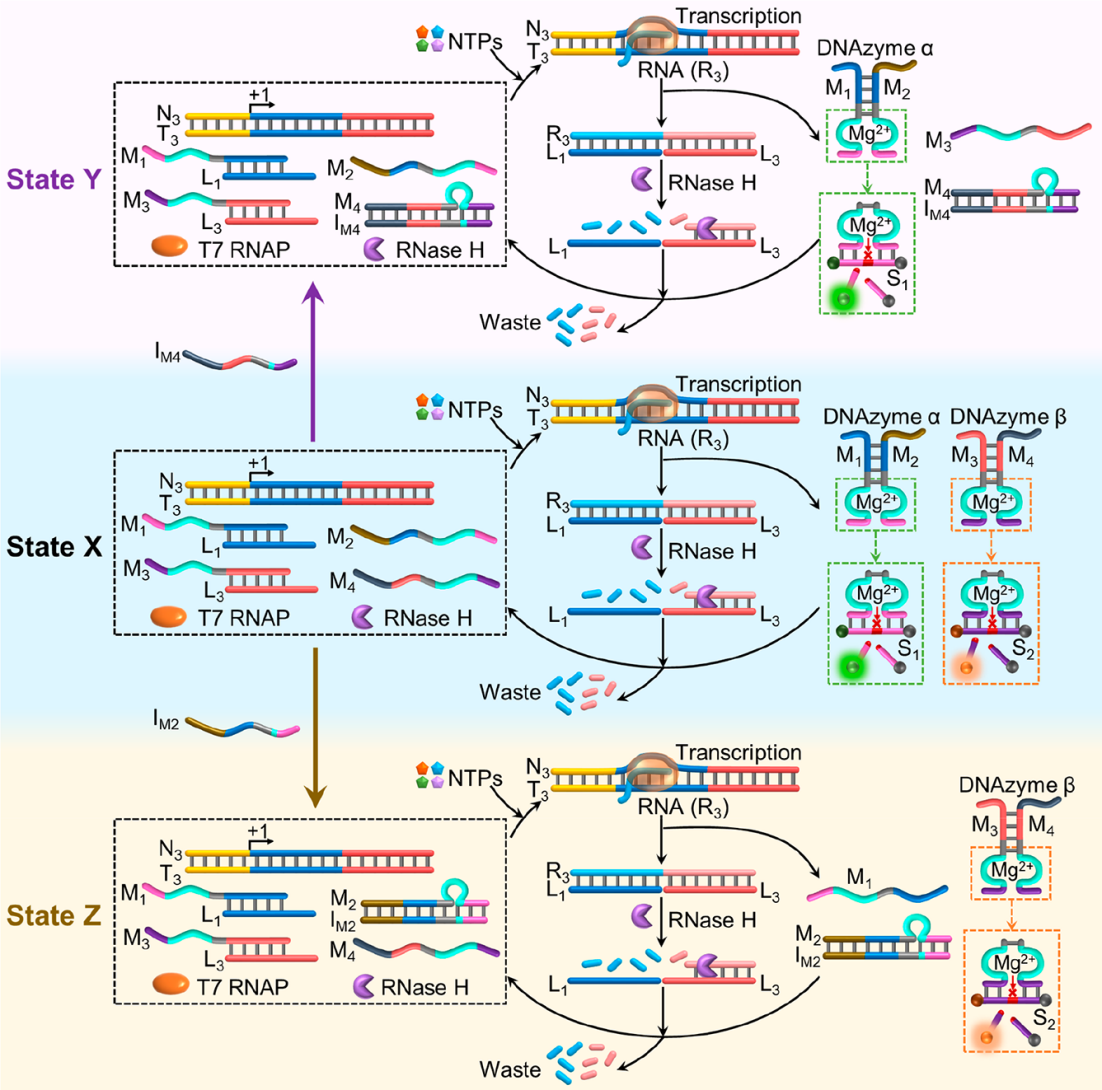
Schematic reaction module driving the gated transient synthesis of DNAzyme α and/or DNAzyme β, using a common transcription machinery. The gating mechanism involves the application of inhibitor I_M_4__, which blocks constituent M_4_ to yield the reaction module in state Y that dictates the selective formation of DNAzyme α, or the application of I_M_2__, which blocks constituent M_2_ to yield the reaction module in state Z that dictates the selective formation of DNAzyme β.

The successful operation of the transient formation and dissipative depletion of a catalytic DNAzyme agent by means of a fuel-triggered reaction module that activates a transcription machinery was then extended to develop a gated transcription machinery that guides the operation of two DNAzymes (Figure 2). The reaction module is composed of transcription template N_3_/T_3_, two caged duplexes M_1_/L_1_ and M_3_/L_3_ and two single strands M_2_ and M_4_, and the enzymes T7 RNAP and RNase H. The strands M_1_, M_2_, M_3_, and M_4_ are engineered to act as functional Mg^2+^-DNAzyme subunits to assemble two different Mg^2+^-ion-dependent DNAzymes, M_1_/M_2_ (DNAzyme α) and M_3_/M_4_ (DNAzyme β) that differ in their substrate-recognition areas. The NTPs-fueled triggering of the reaction module activates the transcription machinery, resulting in the template-mediated transcription of RNA (R_3_) that includes complementary recognition sequences for L_1_ and L_3_. The transcribed R_3_ displaces the duplex M_1_/L_1_ and M_3_/L_3_, resulting in the formation of the bifunctional duplex R_3_/L_1_+L_3_ and the release of the M_1_ and M_3_ strands. While the released strands M_1_ and M_3_ self-assemble into two Mg^2+^-ion-dependent DNAzymes, M_1_/M_2_, M_3_/M_4_, the resulting R_3_/L_1_+L_3_ is “digested” by RNase H to yield the R_3_ “waste” products and the separated strands L_1_ and L_3_. The latter two strands separate the transient DNAzyme structures to recover the rest reaction module. Thus, the NTPs-fueled activation of the reaction module activates the transcription machinery that yields two transient DNAzyme intermediates that are depleted by the degradation of the transcribed RNA. The transient formation of the system was then quantitatively probed by the cleavage of the substrates F_1_/Q_1_-modified substrate S_1_ and the F_2_/Q_2_-modified substrate S_2_, activated by the two DNAzymes α and β (Figure S13).

**Figure 3:**
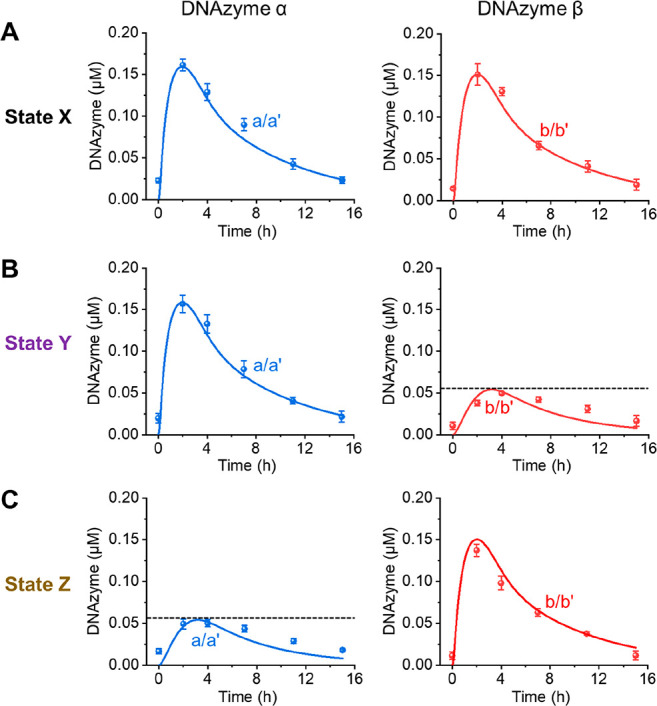
Transient concentrations of DNAzyme α and DNAzyme β, generated by the gated reaction module shown in Figure 2: (A) state X, (B) state Y in the presence of I_M_4__ = 2 μM, and (C) state Z in the presence of I_M_2__ = 2 μM. Curves a and b (dotted) correspond to experimental data of transient concentrations of DNAzyme α and DNAzyme β, respectively, and curves a′ and b′ (solid) correspond to computationally simulated results using the kinetic model formulated in Figures S14–S16. The composition of the reaction module includes: N_3_/T_3_ = 0.2 μM, M_1_/L_1_ = 0.5 μM, M_2_ = 0.5 μM, M_3_/L_3_ = 0.5 μM, M_4_ = 0.5 μM, NTPs = 1 mM, T7 RNAP = 3 U/μL (0.048 μM), RNase H = 8 U/mL (0.266 nM). Error bars represent standard deviations of three measurements.

Figure 3A depicts the concomitant transient operation of DNAzyme α and DNAzyme β in the system. The gated, transcription-guided, selective assembly of one of the DNAzymes is achieved by the introduction of an inhibitor strand that selectively blocks one of the DNAzyme subunits in the reaction module. For example, subjecting the reaction module to inhibitor I_M_4__, which blocks the subunit M_4_ of DNAzyme β, yields state Y where the NTPs-triggered activation of the machinery activates the transcription of R_3_ that displaces the duplex M_1_/L_1_ and M_3_/L_3_ and leads to the hybridization of the L_1_ and L_3_ with R_3_. The caged structure M_4_/I_M_4__ inhibits, however, the self-assembly of DNAzyme β (composed of M_3_/M_4_), resulting in the selective formation of DNAzyme α (composed of M_1_/M_2_). The duplex R_3_/L_1_+L_3_ is cleaved by RNase H, resulting in the transient dissipative separation of DNAzyme α. The gated transient operation of the intermediate DNAzyme α in the system and the concomitant blocked configuration of DNAzyme β are depicted in Figure 3B, showing the transient activities of DNAzyme α (following the cleavage of S_1_ by samples withdrawn, at time intervals, from the reaction mixture in state Y). While DNAzyme α reveals the transient catalytic activities, the catalytic functions of DNAzyme β are blocked. Similarly, subjecting the reaction module in state X with the inhibitor I_M_2__ transforms state X into state Z, where stand M_2_ (subunit of DNAzyme α) is blocked. Under these conditions, the NTPs-triggered activation of the transcription machinery leads to the gated transient formation of DNAzyme β, where the catalytic functions of DNAzyme α are blocked. The transient formation of DNAzyme β is, then, probed by the temporal catalytic cleavage of substrate S_2_ at time intervals of the operation of the system. Figure 3C depicts the gated transient catalytic functions of DNAzyme β, whereas the catalytic functions of DNAzyme α are blocked (Figure 3C) in state Z of the system. It should be noted that even in the presence of excesses of the inhibitors I_M_4__ and I_M_2__, residual contents of the DNAzymes β and α are detected. Presumably, these residues are generated by the displacement of the hybrid duplex M_4_/I_M_4__ or M_2_/I_M_2__ by the free strands M_3_ or M_1_ generated concomitantly by the transcription machinery-stimulated separation of the duplexes M_3_/L_3_ or M_1_/L_1_, respectively. The transient behavior of the gated reaction module was computationally simulated by applying the rate constants derived for the single DNAzyme α, while adapting the kinetic scheme to the coparticipation of DNAzyme β and introducing additional rate constants corresponding to the blocking of M_2_ by I_M_2__ and of M_4_ by I_M_4__. The resulting kinetic models are summarized in Figures S14–S16. The simulated curves corresponding to DNAzyme α–a′ and DNAzyme β–b′ in the different states of the gated module are overlaid on the respective experimental transient curves a and b, and the derived rate constants for the gated transient operation of the DNAzymes α and β are summarized in Tables S4–S6. The significance of these sets of rate constants rests on the ability to predict the gating efficiency of the system at variable concentrations of the blockers (for example, see Figure S17).

### A Transient Catalytic DNAzyme Cascade Operated by Interconnected Dynamic Transcription Machineries

**Figure 4:**
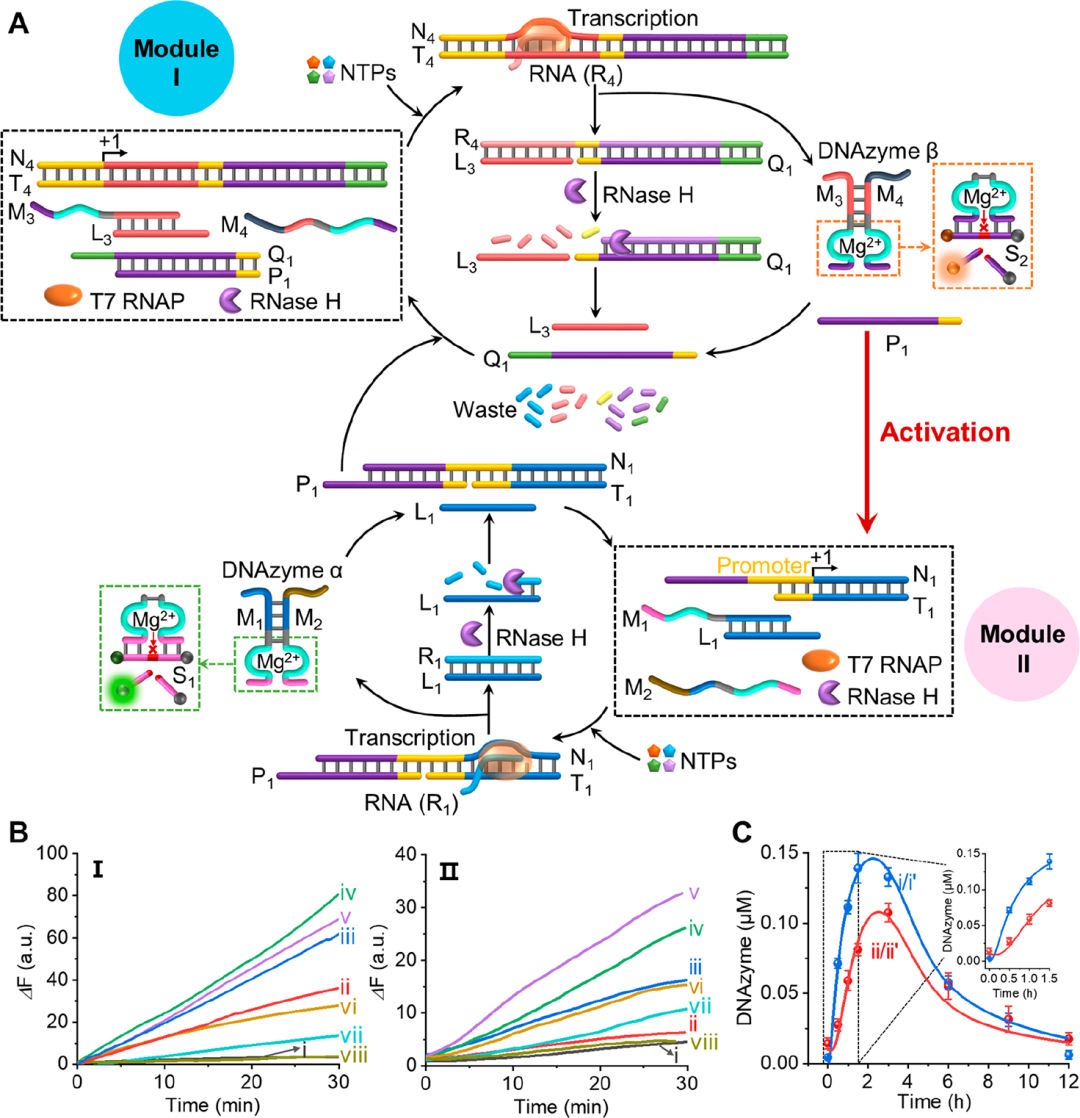
(A) Schematic network corresponding to the intercommunication of two reaction modules, module I and module II, leading to the transient cascaded synthesis of DNAzyme β and DNAzyme α driven by two coupled transcription machineries. (B) Time-dependent fluorescence changes generated by the DNAzymes samples extruded at time intervals of operation of the cascaded network displayed in (A), upon cleavage of the fluorophore/quencher-functionalized substrates of DNAzyme β (panel I) and DNAzyme α (panel II). (DNAzyme α cleaves FAM/BHQ1-modified substrate S_1_ and DNAzyme β cleaves ROX/BHQ2-modified substrate S_2_.) The time intervals at which the samples were withdrawn from the system corresponded to (i) *t* = 0, (ii) 0.5 h, (iii) 1 h, (iv) 1.5 h, (v) 3 h, (vi) 6 h, (vii) 9 h, and (viii) 12 h. (C) Temporal concentrations of DNAzyme β (curve i/i′) and DNAzyme α (curve ii/ii′). Curves i and ii, dotted transients, correspond to experimental concentrations of the respective DNAzymes. Curves i′ and ii′, solid transients, correspond to computationally simulated results. The experimental concentrations of the DNAzymes were evaluated by deriving calibration curves relating to the cleavage rates of substrates S_1_ and S_2_ using variable concentrations of DNAzyme α and DNAzyme β and applying the temporal rates shown in (B), panel I and panel II, to extract the temporal concentrations of the DNAzymes. The computationally simulated concentrations of DNAzyme α and DNAzyme β were evaluated by using the kinetic model formulated in Figure S18. The composition of the reaction module includes: N_4_/T_4_ = 0.2 μM, N_1_/T_1_ = 0.2 μM, P_1_/Q_1_ = 0.5 μM, M_1_/L_1_ = 0.5 μM, M_2_ = 0.5 μM, M_3_/L_3_ = 0.5 μM, M_4_ = 0.5 μM, NTPs = 1 mM, T7 RNAP = 3 U/μL (0.048 μM), RNase H = 10 U/mL (0.332 nM). Error bars represent standard deviations of three measurements.

The transcription machineries were further applied to operate a transient catalytic DNAzyme cascade (Figure 4A). The system consists of two reaction modules. Module I includes the transcription template N_4_/T_4_, the caged duplex M_3_/L_3_, and the free single strand M_4_, where M_3_ and M_4_ correspond to the subunits of DNAzyme β. The enzymes T7 RNAP and RNase H are also included in the reaction module. In addition, a duplex P_1_/Q_1_ is included in the module, and it acts as a key functional element that intercommunicates between modules I and II. Module II includes an incomplete, inactive, transcription template, N_1_/T_1_, the caged duplex M_1_/L_1_, and the single strand M_2_, where M_1_ and M_2_ correspond to the subunits of DNAzyme α, and the enzyme T7 RNAP and RNase H. In the presence of the NTPs as fuel, the transcription machinery associated with module I is triggered “ON” to yield R_4_. The transcribed R_4_ displaces the duplex M_3_/L_3_ and the duplex P_1_/Q_1_, which separates the duplexes to yield the single strands M_3_ and P_1_ while stabilizing the newly formed duplex R_4_/L_3_+Q_1_. These chemical events generate DNAzyme β and the duplex R_4_/L_3_+Q_1_ as intermediate products, accompanied by the free strand P_1_. Meanwhile, the R_4_ constituent within the duplex R_4_/L_3_+Q_1_ is cleaved by RNase H to yield the fragmented R_4_ as “waste” and to separate the L_3_ and Q_1_ as transient constituents. The released L_3_ and Q_1_ separate the DNAzyme β and capture P_1_, respectively, to restore the rest state of module I. This set of dynamic reactions leads to the transient formation and depletion of DNAzyme β, and its temporal activity is monitored by the DNAzyme β-stimulated cleavage of the fluorophore/quencher-modified substrate S_2_. The temporally generated strand P_1_ is, however, tailored to hybridize with the incomplete transcription module N_1_/T_1_ being a part of module II. The integration of P_1_ within N_1_/T_1_ assembles the intact transcription template N_1_/T_1_+P_1_, resulting in the activation of the transcription machinery in module II, yielding R_1_. The transcribed R_1_ separates the duplex M_1_/L_1_, generates the duplex R_1_/L_1_, and guides the assembly of DNAzyme α composed of M_1_/M_2_. The products M_1_/M_2_ and R_1_/L_1_ act, however, as intermediate agents, where R_1_ is cleaved by RNase H to yield “waste” products separating L_1_, resulting in the separation of DNAzyme α to form the M_1_/L_1_ duplex. Meanwhile, the P_1_-guided assembly of the active transcription template of module II reveals transient, intermediate features. Its displacement by Q_1_, generated temporally in module I, regenerates the inactive template N_1_/T_1_ associated with module II, and together with the transient, generated M_1_/L_1_ duplex restores the rest module II, and completes the recovery of the rest module I. The conjugation of the two modules leads to the activation of the formation of a transiently operating cascade of two DNAzymes, DNAzyme β and DNAzyme α. The transient operation of the two DNAzymes was then probed by sampling the catalytic activities of DNAzyme β and DNAzyme α along the temporal operation of the cascaded catalytic system.

Figure 4B depicts the time-dependent fluorescence changes corresponding to the rates of cleavage of substrate S_2_ by DNAzyme β, panel I, and the rates of cleavage of substrate S_1_ by DNAzyme α, panel II, from samples extruded at time intervals from the two-module reaction mixture. Using appropriate calibration curves relating to the rates of cleavage of substrates S_1_ and S_2_ by variable concentrations of the DNAzymes (Figure S1), quantitative temporal concentrations of the DNAzymes were assessed, and these are displayed in Figure 4C, curves i and ii, respectively. The two cascaded DNAzymes reveal transient formation and depletion behaviors. The kinetics of the two-enzyme cascade was computationally simulated. A kinetic model for the system was formulated (Figure S18) where additional subreactions associated with the coupling between the two moduli were integrated into the set of subreactions associated with DNAzyme β and DNAzyme α. Using the kinetic model, the computationally simulated kinetic behavior of the DNAzymes are overlaid the experimental results, curves i′ and ii’′ (solid curves, Figure 4C). The derived rate constants for the set of subreactions associated with the cascaded network are summarized in Table S7. Several important features regarding the cascaded temporal kinetics of DNAzyme β and DNAzyme α should be mentioned: (i) The activation of module II by the module I-generated promoter P_1_ suggests that the temporal accumulation of P_1_ will introduce a lag time interval in the transient operation of DNAzyme α generated by the transcription machinery of module II. Indeed, the inset of Figure 4C depicts the temporal kinetics of DNAzyme β and DNAzyme α at a short time interval of the temporal operation of the two DNAzymes. Obviously, DNAzyme α shows a short lag period of ca. 20 min and a delay in its peak content as compared to DNAzyme β. This result is consistent with the temporal accumulation of P_1_ for operating module II. (ii) The cascaded operation of DNAzyme β and DNAzyme α shows a higher temporal activity of DNAzyme β as compared to DNAzyme α. Furthermore, the activity of DNAzyme α in the cascaded process is lower than the temporal activity of DNAzyme α in the single module construct (Figure 1C, panel II). Two reasons might cooperatively contribute to the lower temporal activity of DNAzyme α. One reason is associated with the supply of promoter P_1_ by module I to activate the transcription machinery of module II. As the temporal operation of module I involves the RNase H depletion of P_1_, the supply of P_1_ to module II is dampened by the temporal behavior of module I. A second reason for the lower activity of DNAzyme α in the cascaded system might originate from the higher concentration of RNase H used to operate the cascaded system (10 U/mL) as compared to the concentration of RNase H in the single module system generating DNAzyme α (Figure 1C, 6 U/mL). The higher concentration of RNase H depletes the intermediate DNAzyme α faster, leading to a lower content of this catalyst. A further control experiment involving a modified template in module I has emphasized the key interrelationship between modules I and II that is needed to operate the two-layer cascade (for explanation of this control experiment, see Figure S19 and accompanying discussion, and for computationally simulated kinetic model and the derived rate constants, see Figure S20 and Table S8).

## Conclusion

The study has introduced a versatile concept to emulate native networks where transient transcription machineries modulate transient, dissipative, cellular processes. The concept is based on the design of reaction modules consisting of transcription templates, caged and inactive nucleic acid constituents exhibiting structural ability to assemble into catalytically active DNAzyme structures. The reaction modules include two enzymes, T7 RNAP and RNase H, as catalytic agents that control the dynamic modulation of the reaction modules. In the presence of the nucleotide bases, NTPs, as fuel, the transcription machineries are activated toward the RNAP-catalyzed synthesis of intermediate products that displace the caged constituents and allow the self-assembly of catalytically active Mg^2+^-ion-dependent DNAzyme units. The concomitant RNase-stimulated digestion of the RNA products releases, however, the free caging constituents, resulting in the dissociation of the self-assembled DNAzyme units and the recovery of the parent muted reaction modules. Accordingly, the systems mimic biological dissipative reaction pathways where the energy input—the NTPs fuel—activates the transient formation of intermediate catalysts, through the transcription-guided synthesis of RNA, that is degraded into “waste” products while allowing the temporal operation of the DNAzyme catalysts. The transient operation of the DNAyzmes is probed by the temporal DNAzyme-catalyzed cleavage of the respective fluorophore/quencher-functionalized substrates. By designing different reaction modules, gated, transcription machinery-guided transient operation of two different DNAzymes is demonstrated. In addition, by intercommunication of two functional reaction modules, the transcription-guided cascaded transient operation of two DNAzymes is accomplished. In fact, recent reports discussed different methods to modulate transcription machineries by artificial means. These included the temporal transcription-guided assembly and RNase-induced disassembly of microtubes^55^ or aggregated nanoparticles,^57^ the transcription-controlled temporal ligation and nicking of intercommunicated DNA assemblies,^60^ and the transcription machinery temporal modulated synthesis of a ribozyme through the transient inhibition of T7 RNAP by a transcribed aptamer.^58^ Thus, the advantages and advancements introduced by the present study should be addressed. While the RNase-modulated transcription-controlled formation and depletion of DNA microstructures or particle aggregates^55,57,60^ did not lead to any temporal catalytic outputs, only the transcription-inhibited T7 RNAP system led to a catalytic ribozyme output.^58^ Nonetheless, in this later system, the transient formed ribozyme is accumulated in the system, in contrast to the transcription/RNase-modulated depletion of the DNAzymes formed in the present study. The depletion of the transcription-modulated DNAzymes is particularly important in view of the envisaged medical applications of such transient DNAzyme agents. The transient formation and depletion of DNAzymes is envisaged to act as a temporal treatment agent of a medical event, and thus the accumulation of the catalyst should be prevented. Beyond the combined experimental and computational kinetically modeled transcription circuitries presented in our study, our results contribute a possible future medical application of the systems.

An important facet of the study is the kinetic modeling of the complex dissipative transformations. The formulation of kinetic models and the computational simulation of the rate constants of the subreactions involved in these temporal dissipative systems is suggested as a key step for understanding the systems and as versatile method that should be adopted to quantitatively analyze dynamic networks. The computational simulations of the kinetics of dynamic networks not only provide rate constants that quantitatively account for the subreactions associated with the networks but also introduce tools to predict the behavior of the networks under different auxiliary conditions that can be later experimentally validated.

Beyond the significance of the present study in advancing the fields of systems chemistry and synthetic biology and demonstrating transcription machineries as functional tools that control dynamic transient catalysis, the future perspectives should be mentioned. The energy-fueled synthesis of programmed and controlled catalysts at the expense of the generation of “waste” products is the primary step of a living system. Albeit the present systems operate in homogeneous aqueous phases, the integration of such systems into cell-like containments, such as vesicles,^61,62^ polymersomes,^63,64^ dendrosomes,^65^ or hydrogel microcapsules,^66^ protocells,^11,12^ could yield simple biomimetic models for dynamically controlled cellular transformations.

## Experimental Section

### Nucleotide Mixture-Fueled Activation of the Transcription Machinery-Guided Transient Operation of a DNAzyme

To prepare the transcription machinery-guided operation of a transient DNAzyme shown in Figure 1, N_1_/T_1_+P_1_ (10 μM) and M_1_/L_1_ (25 μM) were annealed in 1 × RNAPol reaction buffer, respectively, at 90 °C for 5 min and cooled down to 25 °C over 30 min. The reaction mixture (800 μL) consisting of N_1_/T_1_+P_1_ (0.2 μM), M_1_/L_1_ (0.5 μM), and M_2_ (0.5 μM) in 1 × RNAPol reaction buffer (supplemented with 5 mM DTT and 10 mM MgCl_2_) was subjected to variable concentrations of NTPs, T7 RNAP, and RNase H, then incubated at 33 °C.

### Gated, Transcription Machinery-Guided Operation of Two Dissipative DNAzymes

To prepare the gated, transcription machinery-guided operation of two DNAzymes (state X, nongated) shown in Figure 2, N_3_/T_3_ (10 μM), M_1_/L_1_ (25 μM), and M_3_/L_3_ (25 μM) were annealed in 1 × RNAPol reaction buffer, respectively, at 90 °C for 5 min and cooled down to 25 °C over 30 min. A reaction mixture (1.2 mL) consisting of N_3_/T_3_ (0.2 μM), M_1_/L_1_ (0.5 μM), M_3_/L_3_ (0.5 μM), M_2_ (0.5 μM), M_4_ (0.5 μM), T7 RNAP (3 U/μL), RNase H (8 U/mL), and NTPs (1 mM) in 1 × RNAPol reaction buffer (supplemented with 5 mM DTT and 10 mM MgCl_2_) was incubated at 33 °C. For the inhibitor I_M_4__ or I_M_2__-gated, transcription machinery-guided operation of two DNAzymes (state Y or Z), reaction mixtures (1.2 mL) consisting of N_3_/T_3_ (0.2 μM), M_1_/L_1_ (0.5 μM), M_3_/L_3_ (0.5 μM), M_2_ (0.5 μM), M_4_ (0.5 μM), T7 RNAP (3 U/μL), RNase H (8 U/mL), and NTPs (1 mM) in 1 × RNAPol reaction buffer (supplemented with 5 mM DTT and 10 mM MgCl_2_), were subjected to the inhibitor I_M_4__ or I_M_2__ (2 μM), followed by incubation at 33 °C.

### Cascaded Operation of Two Transient DNAzymes Driven by Two Interconnected Dynamic Transcription Machineries

To prepare the cascaded transcription machinery-guided operation of DNAzymes shown in Figure 4, N_4_/T_4_ (10 μM), N_1_/T_1_ (10 μM), M_1_/L_1_ (25 μM), M_3_/L_3_ (25 μM), and P_1_/Q_1_ (25 μM) were annealed in 1 × RNAPol reaction buffer, respectively, at 90 °C for 5 min and cooled down to 25 °C over 30 min. A reaction mixture (1.2 mL) consisting of N_4_/T_4_ (0.2 μM), N_1_/T_1_ (0.2 μM), M_1_/L_1_ (0.5 μM), M_3_/L_3_ (0.5 μM), M_2_ (0.5 μM), M_4_ (0.5 μM), P_1_/Q_1_ (0.5 μM), T7 RNAP (3 U/μL), RNase H (10 U/mL), NTPs (1 mM) in 1 × RNAPol reaction buffer (supplemented with 5 mM DTT and 10 mM MgCl_2_) was subjected to then incubated at 33 °C. For the negative control of the cascaded DNAzymes system shown in Figure S19, a reaction mixture (1.2 mL) consisting of N_5_/T_5_(0.2 μM), N_1_/T_1_ (0.2 μM), M_1_/L_1_ (0.5 μM), M_3_/L_3_ (0.5 μM), M_2_ (0.5 μM), M_4_ (0.5 μM), P_1_/Q_1_ (0.5 μM), T7 RNAP (3 U/μL), RNase H (10 U/mL), NTPs (1 mM) in 1 × RNAPol reaction buffer (supplemented with 5 mM DTT and 10 mM MgCl_2_) was incubated at 33 °C.

### Probing the Activity of Transiently Operating DNAzymes

For each transcription machinery-guided dissipative DNAzyme system prepared, aliquots of 100 μL were withdrawn at time intervals and treated with 1 μL of fluorophore/quencher-modified substrate S_1_ or S_2_ stock solution (100 μM). The time-dependent fluorescence changes (λ_ex_ = 496 nm, λ_em_ = 520 nm) generated by the cleavage of FAM/BHQ1-modified substrate S_1_ by DNAzyme α and/or fluorescence changes (λ_ex_ = 588 nm, λ_em_ = 608 nm) generated by the cleavage of ROX/BHQ2-modified substrate S_2_ by DNAzyme β were monitored on a Cary Eclipse Fluorometer (Varian Inc.) at 25 °C using plastic cuvettes with 10 mm path lengths. The temporal concentrations of the DNAzymes were quantified by using the appropriate calibration curves relating to the catalytic rates (d(Δ*F*)/d*t*) of cleavage of substrates by variable standard concentrations of Mg^2+^-ion-dependent DNAzymes.

## Supporting Information Available

Chemicals, oligonucleotide sequences, transcription-guided synthesis of malachite green aptamers, real-time fluorescence monitoring of the RNA transcript-mediated strand displacement, calibration curves corresponding to DNAzyme activities, DNAzyme activities in the absence of NTPs fuels, operation of two cycles of the DNAzyme α system, the effect of RNase H on DNAzyme substrate, optimization of the DNA template concentrations, computational kinetic models and discussions for simulations of the transcription machineries-guided synthesis of the temporally operating DNAzymes, gated and cascaded DNAzyme catalysis, control experiment for the two-layer cascaded DNAzyme system, tables summarizing the rate constants derived from the computational simulation (PDF)
