# Supplementary material for: Dynamic Transcription Machineries Guide the Synthesis of Temporally Operating DNAzymes, Gated and Cascaded DNAzyme Catalysis
Source: ACS Nano. 2022 Dec 28;17(1):687–96. doi: 10.1021/acsnano.2c10108 (PMC9836355; doi:10.1021/acsnano.2c10108)
Supplement: Supplementary file 1 — nn2c10108_si_001.pdf [file nn2c10108_si_001.pdf]

Supporting Information

Dynamic Transcription Machineries Guide the Synthesis of Temporally Operating DNAzymes, Gated and Cascaded DNAzyme Catalysis

*Jiantong Dong, Itamar Willner**

Institute of Chemistry, Center for Nanoscience and Nanotechnology, The Hebrew University of Jerusalem, Jerusalem 91904, Israel.

*Email: itamar.willner@mail.huji.ac.il .

Table of Contents:

1. Supplemental Experimental Section

2. Supplemental Figures 1–20 and Discussions

3. Supplemental Tables 1–8

4. Supplemental References

1. Supplemental Experimental Section

Chemicals

DNA oligonucleotides, fluorophore and quencher-modified substrates, RNA, and nuclease-free water were purchased from Integrated DNA Technologies Inc. T7 RNA polymerase (50,000 units/mL, 0.8 μM), RNase H (5,000 units/mL, 0.166 μM), ribonucleotide (NTP) Mix (GTP, ATP, UTP and CTP, each 25 mM), and 10 × RNAPol reaction buffer (400 mM Tris-HCl, 60 mM $MgCl_2$, 10 mM DTT, 20 mM spermidine, pH 7.9 @ 25 °C) were purchased from New England BioLabs Inc. Malachite Green (MG), DTT, and $MgCl_2$ were purchased from Sigma-Aldrich.

The oligonucleotide sequences used in the study (5′→3′)[a]:

N$_1$: ATTGAGGTAAGAAAGGTAAGGATAATACGACTCACTATAGGGATTGAGC GTTCTTCTGTC

T$_1$: GACAGAAGAACGCTCAATCCCTATAGTGAGTCG

N$_2$: ATTGAGGTAAGAAAGGTAAGGATAATACGACTCACTATAGGGATGGCCT CAGCGTTCTTCTGTC

T$_2$: GACAGAAGAACGCTGAGGCCATCCCTATAGTGAGTCG

N$_3$: TAATACGACTCACTATAGGGATTGAGCGTTCTTCTGTCTGTAGTAGTCAG CCAGTGGAA

T$_3$: TTCCACTGGCTGACTACTACAGACAGAAGAACGCTCAATCCCTATAGTG AGTCGTATTA

N$_4$: TAATACGACTCACTATAGGGGTAGTAGTCAGCCAGTGGAAATTATCCTTA CCTTTCTTACCTCAATCTTCGCCTTCTTACCTG

T$_4$: CAGGTAAGAAGGCGAAGATTGAGGTAAGAAAGGTAAGGATAATTTCCA CTGGCTGACTACTACCCCTATAGTGAGTCGTATTA

N$_5$: TAATACGACTCACTATAGGGGTAGTAGTCAGCCAGTGGAA

T$_5$: TTCCACTGGCTGACTACTACCCCTATAGTGAGTCGTATTA

P$_1$: TATTATCCTTACCTTTCTTACCTCAATCTTCGCCT

Q$_1$: CAGGTAAGAAGGCGAAGATTGAGGTAAGAAAGGTAAGGATAATA

L_1: ACAGAAGAACGCTCAATCCTTG

Cy3-labeled L_2: Cy3-GACAGAAGAACGCTGAGGCCATC

BHQ2-labeled L_2': GATGGCCTCAGCGTT-BHQ2

D_2: GGGATGGCCTCAGCGTTCTTCTGTC

L_3: TTCCACTGGCTGACTACTACCAC

M_1: *CCATTCAGCGAT*AGACAAGGATTGAGCGTT

M_2: GAACCGTTTGAATCCTTGTCT*CACCCATGTTTAGCT*

M_3: *GTCCTCAGCGAT*CAAGTGGTAGTAGTCAGCC

M_4: TTCGTCCTACACTACCACTTG*CACCCATGTTCCTGA*

I_{M2}: AGCTAAACTAGACAAGGATTCAAACGGTTC

I_{M4}: TCAGGAACTCAAGTGGTAGTGTAGGACGAA

S_1: FAM-AGCTAAT**rA**GGAATGG-BHQ1

S_2: ROX-TCAGGAT**rA**GGAGGAC-BHQ2

N_{MG}: CTAATGAACTACTACTGATCACTAATACGACTCACTATAGGGATCCCGACTGGCGAGAGCCAGGTAACGAATGGATCC

T_{MG}: GGATCCATTCGTTACCTGGCTCTCGCCAGTCGGGATCCCTATAGTGAGTCG

P_2: TATTAGTGATCAGTAGTAGTTCATTAGTGTCGTTC

MG aptamer: GGAUCCCGACUGGCGAGAGCCAGGUAACGAAUGGAUCC

[a] The promoter sequences of DNA templates are underlined. The Mg^{2+}-ion-dependent DNAzyme sequences are shown in italics. The ribonucleobase cleavage site rA in substrates S_1 and S_2 is presented in bold.

Transcription-guided synthesis of MG aptamers

For fluorescence monitoring of transcription-guided synthesis of the MG aptamer shown in Figure S6, $N_{MG}/T_{MG}+P_2$ (10 μM) was annealed in 1 × RNAPol reaction buffer at 90 °C for 5 min and cooled down to 25 °C over 30 min. The reaction mixture (100 μL) consisting of $N_{MG}/T_{MG}+P_2$ (0.2 μM), MG (5 μM), NTPs (0.5 mM), and T7 RNAP (2 U/μL), in 1 × RNAPol reaction buffer (supplemented with 5 mM DTT and 10 mM $MgCl_2$), was incubated at 33 °C. Time-dependent fluorescence changes (λ_{ex} = 632 nm,

λ_{em} = 650 nm) resulting from the binding of transcribed RNA to MG were monitored on a Cary Eclipse Fluorometer (Varian Inc) at 33 °C using plastic cuvettes with 10-mm path lengths. The concentration of the transcribed RNA was quantified by using the appropriate calibration curves corresponding to changes in MG fluorescence upon adding different standard concentrations of MG aptamer.

Real-time fluorescence monitoring of the transient transcription machinery

For real-time fluorescence monitoring of the transcription machinery shown in Figure S7, N_2/T_2+P_1 (10 μM) and Cy3-labeled L_2/BHQ2-labeled L_2' (25 μM) were annealed in 1 × RNAPol reaction buffer, respectively, at 90 °C for 5 min and cooled down to 25 °C over 30 min. The reaction mixture (100 μL) consisting of N_2/T_2+P_1 (0.2 μM), Cy3-labeled L_2/BHQ2-labeled L_2' (0.5 μM), T7 RNAP (3 U/μL), and NTPs (0.5 mM), in 1 × RNAPol reaction buffer (supplemented with 5 mM DTT and 10 mM $MgCl_2$), was incubated at 33 °C.

For real-time fluorescence monitoring of the dissipative transcription machinery shown in Figure S9, reaction mixtures (each 100 μL) consisting of N_2/T_2+P_1 (0.2 μM), Cy3-labeled L_2/BHQ2-labeled L_2' (0.5 μM) and T7 RNAP (2 U/μL), in 1 × RNAPol reaction buffer (supplemented with 5 mM DTT and 10 mM $MgCl_2$), were subjected to variable concentrations of NTPs and RNase H, then incubated at 33 °C.

Time-dependent fluorescence changes (λ_{ex} = 540 nm, λ_{em} = 564 nm) generated by the displacement of Cy3-labeled L_2/BHQ2-labeled L_2' by the transcribed RNA (R_2) were monitored on a Cary Eclipse Fluorometer (Varian Inc) at 33 °C using plastic cuvettes with 10-mm path lengths. The temporal concentrations of the displaced Cy3-labeled L_2 were quantified by using the appropriate calibration curves corresponding to the fluorescence changes of Cy3-labeled L_2/BHQ2-labeled L_2' upon adding different standard concentrations of D_2.

2. Supplemental Figures and Discussions

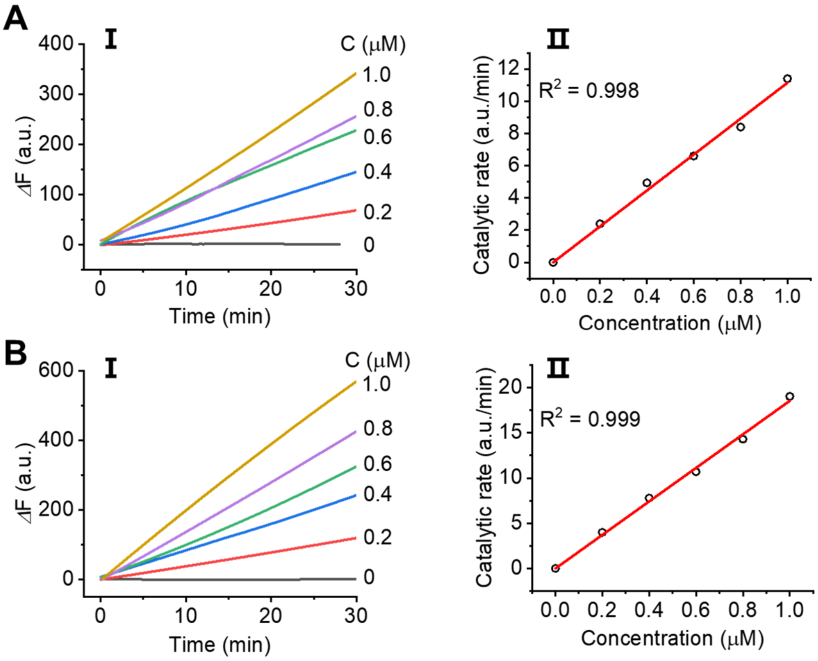

Figure S1. (A) Panel I: Time-dependent fluorescence changes generated from the cleavage of FAM/BHQ1-modified substrate S_1 by variable concentrations of Mg^{2+}-ion-dependent DNAzyme α (M_1/M_2). Panel II: The derived calibration curve relating to the rates of cleavage of S_1 by different concentrations of DNAzyme α. (B) Panel I: Time-dependent fluorescence changes generated from the cleavage of ROX/BHQ2-modified substrate S_2 by variable concentrations of Mg^{2+} -ion-dependent DNAzyme β (M_3/M_4). Panel II: The derived calibration curve relating to the rates of cleavage of S_2 by different standard concentrations of DNAzyme β.

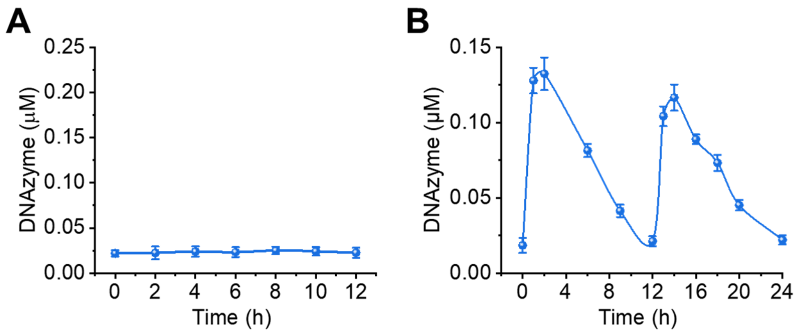

Figure S2. (A) Transient concentrations of DNAzyme α in the absence of added NTPs during the time interval of the experiment. (B) Operation of two cycles of the systems depicted in Figure 1 upon re-addition of a new batch of NTPs (0.5 mM) after the first transient cycle. Reaction conditions are $N_1/T_1+P_1 = 0.2$ μM, $M_1/L_1 = 0.5$ μM, $M_2 = 0.5$ μM, T7 RNAP = 2 U/μL (0.032 μM), and RNase H = 6 U/mL (0.199 nM).

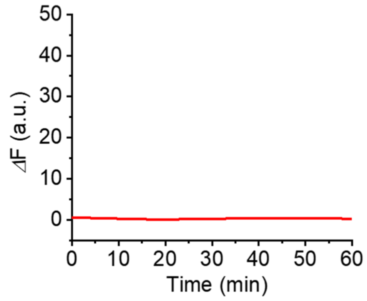

Figure S3. RNase H does not hydrolysis the DNAzyme substrate. Reaction conditions are RNase H = 6 U/mL (0.199 nM), DNAzyme substrate $S_1 = 1$ μM, 1 × RNAPol reaction buffer, 25 °C.

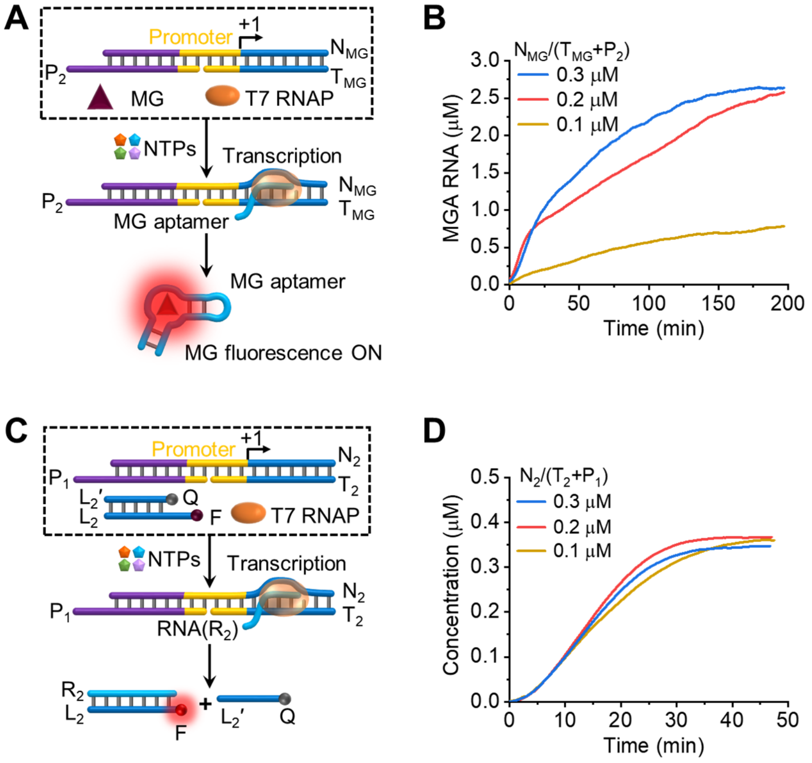

Figure S4. Optimization of the concentrations of the DNA templates for operation of transcription machineries. (A) Schematic illustration of the transcription-guided synthesis of MG RNA aptamer, which lights up MG fluorescence for monitoring the transcription process. (B) The dynamic concentration of the transcribed MG RNA aptamer under different concentrations of DNA template $N_{MG}/(T_{MG}+P_2)$: 0.1 μM, 0.2 μM, and 0.3 μM, using the calibration curves shown in the Figure S6C. Conditions are MG = 5 μM, T7 RNAP = 2 U/μL, NTPs = 0.5 mM, 33 °C. (C) Schematic illustration of a real-time transcription machinery, which is triggered by NTPs to produce RNA (R_2) to displace Cy3-labeled L_2 from the Cy3-labeled L_2/BHQ2-labeled L_2' duplex and thus recover the Cy3 fluorescence. (D) The dynamic concentration of the displaced Cy3-labeled L_2 under different concentrations of DNA template $N_2/(T_2+P_1)$: 0.1 μM, 0.2 μM, and 0.3 μM, using the calibration curves shown in the Figure S7C. Conditions are Cy3-labeled L_2/BHQ2-labeled L_2' = 0.5 μM, T7 RNAP = 2 U/μL, NTPs = 0.5 mM, 33 °C.

To evaluate the effect of DNA template concentration on transcription rate and yield, the transcription of MG RNA aptamer (Figure S4A) was monitored under different concentrations of DNA template. As shown in Figure S4B, when the DNA template was increased from 0.1 μM to 0.2 μM, the transcription rate and yield increased significantly. However, when the template concentration continued to increase up to 0.3 μM, the increase in transcription rate and yield was not significant. Furthermore, the effect of DNA template concentrations was investigated by monitoring the transcription

and RNA strand displacement using the Cy3-labeled L_2/BHQ2-labeled L_2' (Figure S4C). As shown in Figure S4D, while 0.2 μM of DNA template performed a faster rate of the transcribed RNA-mediated strand displacement, no big difference was observed among different concentrations of the DNA template. Therefore, a fixed concentration of 0.2 μM DNA template for transcription was used in all the systems.

Kinetic equations of transcription machinery-guided synthesis of the dissipative transiently operating DNAzyme shown in Figure 1:

(1) $N_1T_1 + P_1 \underset{k_{-1}}{\overset{k_1}{\rightleftarrows}} N_1T_1P_1$ (Activation)

(2) $M_1 + L_1 \underset{k_{-2}}{\overset{k_2}{\rightleftarrows}} M_1L_1$

(3) $N_1T_1P_1 + \text{RNAP} \underset{k_{-3}}{\overset{k_3}{\rightleftarrows}} N_1T_1P_1{\bullet}\text{RNAP}$

(4) $N_1T_1P_1{\bullet}\text{RNAP} + \text{NTPs} \xrightarrow{k_4} \text{RNAP} + N_1T_1P_1 + R_1$ (length = 21, $N_{\max}$ (U) = 8)

(5) $R_1 + M_1L_1 \underset{k_{-5}}{\overset{k_5}{\rightleftarrows}} R_1L_1 + M_1$

(6) $M_1 + M_2 \underset{k_{-6}}{\overset{k_6}{\rightleftarrows}} M_1M_2$

(7) $R_1L_1 + \text{RNaseH} \underset{k_{-7}}{\overset{k_7}{\rightleftarrows}} R_1L_1{\bullet}\text{RNaseH}$

(8) $R_1L_1{\bullet}\text{RNaseH} \xrightarrow{k_8} \text{RNaseH} + L_1Waste_1$

(9) $L_1Waste_1 \underset{k_{-9}}{\overset{k_9}{\rightleftarrows}} L_1 + Waste_1$

Derivatives:

$$\frac{dN_1T_1}{dt} = k_{-1}[N_1T_1P_1] - k_1[N_1T_1][P_1]$$

$$\frac{dP_1}{dt} = k_{-1}[N_1T_1P_1] - k_1[N_1T_1][P_1]$$

$$\frac{dN_1T_1P_1}{dt} = k_1[N_1T_1][P_1] - k_{-1}[N_1T_1P_1] - k_3[N_1T_1P_1][\text{RNAP}] + k_{-3}[N_1T_1P_1{\bullet}\text{RNAP}] + k_4[N_1T_1P_1{\bullet}\text{RNAP}][\text{NTPs}] \times Y / N_{\text{Max}}$$

$$\frac{dM_1}{dt} = k_{-2}[M_1L_1] - k_2[M_1][L_1] + k_{-6}[M_1M_2] - k_6[M_1][M_2] + k_5[R_1][M_1L_1] - k_{-5}[R_1L_1][M_1]$$

$$\frac{dL_1}{dt} = k_{-2}[M_1L_1] - k_2[M_1][L_1] + k_9[L_1Waste_1] - k_{-9}[L_1][Waste_1]$$

$$\frac{dM_1L_1}{dt} = k_2[M_1][L_1] - k_{-2}[M_1L_1] - k_5[R_1][M_1L_1] + k_{-5}[R_1L_1][M_1]$$

$$\frac{d\text{RNAP}}{dt} = k_{-3}[N_1T_1P_1{\bullet}\text{RNAP}] - k_3[N_1T_1P_1][\text{RNAP}] + k_4[N_1T_1P_1{\bullet}\text{RNAP}][\text{NTPs}] \times Y / N_{\text{Max}}$$

$$\frac{dN_1T_1P_1{\bullet}\text{RNAP}}{dt} = k_3[N_1T_1P_1][\text{RNAP}] - k_{-3}[N_1T_1P_1{\bullet}\text{RNAP}] - k_4[N_1T_1P_1{\bullet}\text{RNAP}][\text{NTPs}] \times Y / N_{\text{Max}}$$

$$\frac{d\text{NTPs}}{dt} = -k_4[N_1T_1P_1{\bullet}\text{RNAP}][\text{NTPs}]$$

$$\frac{dR_1}{dt} = k_4[N_1T_1P_1{\bullet}\text{RNAP}][\text{NTPs}] \times Y / N_{\text{Max}} - k_5[R_1][M_1L_1] + k_{-5}[R_1L_1][M_1]$$

$$\frac{dR_1L_1}{dt} = k_5[R_1][M_1L_1] - k_{-5}[R_1L_1][M_1] - k_7[R_1L_1][\text{RNaseH}] + k_{-7}[R_1L_1{\bullet}\text{RNaseH}]$$

$$\frac{dM_2}{dt} = k_{-6}[M_1M_2] - k_6[M_1][M_2]$$

$$\frac{dM_1M_2}{dt} = k_6[M_1][M_2] - k_{-6}[M_1M_2]$$

$$\frac{d\text{RNaseH}}{dt} = k_{-7}[R_1L_1\bullet\text{RNaseH}] - k_7[R_1L_1][\text{RNaseH}] + k_8[R_1L_1\bullet\text{RNaseH}]$$

$$\frac{dR_1L_1\bullet\text{RNaseH}}{dt} = k_7[R_1L_1][\text{RNaseH}] - k_{-7}[R_1L_1\bullet\text{RNaseH}] - k_8[R_1L_1\bullet\text{RNaseH}]$$

$$\frac{dL_1Waste_1}{dt} = k_8[R_1L_1\bullet\text{RNaseH}] - k_9[L_1Waste_1] + k_{-9}[L_1][Waste_1]$$

$$\frac{dWaste_1}{dt} = k_9[L_1Waste_1] - k_{-9}[L_1][Waste_1]$$

Figure S5. Computational simulation of transcription machinery-guided synthesis of a transient DNAzyme shown in Figure 1. The kinetic scheme of the sub-reactions associated with the time-dependent concentration changes during the dissipative transitions is summarized in the above equations. Knowing the time-dependent concentration changes of M_1M_2, we computationally simulated the system by using Matlab R2019b. Initial concentrations of the condition: N_1/T_1+P_1 = 0.2 μM, M_1/L_1 = 0.5 μM, M_2 = 0.5 μM, T7 RNAP = 0.032 μM, and RNase H = 0.199 nM, NTPs = 0.5 mM. The derived rate constants are summarized in Table S1.

The RNA yield (Y), *i.e.*, the conversion efficiency of NTPs into RNA, is calculated as the ratio of the obtained RNA product to the limiting input reagent.[1] The limiting input reagent is defined as the most abundant nucleotide in the transcribed RNA. The number of the most abundant nucleotide in the RNA sequence is denoted as $N_{\max}$. One equivalent of the limit input nucleotide is consumed $N_{\max}$ times during the synthesis of one molecule of RNA. Therefore, the RNA yield is calculated according to the following equation:

$$Y = n_{RNA}/(n_{\text{NTP}}/N_{\max}) \times 100\% = C_{RNA}/(C_{\text{NTP}}/N_{\max}) \times 100\% \qquad \text{(i)}$$

The relationship between the kinetics of RNA synthesis and NTP consumption is as follows:

$$\frac{dR}{dt}(\text{synthesis}) = k[\text{template}\bullet\text{RNAP}][\text{NTPs}]/N_{\max} \times Y \qquad \text{(ii)}$$

$$\frac{d\text{NTPs}}{dt} = -\frac{dR}{dt}(\text{synthesis}) \times N_{\max}/Y = -k[\text{template}\bullet\text{RNAP}][\text{NTPs}] \qquad \text{(iii)}$$

The RNA yield (Y) value was calculated by the transcription-guided synthesis of MG aptamer as shown in Figure S6 under the same experimental conditions.

Kinetic simulation of experimental results

Kinetic models for all the transient systems described in the study were formulated. The initial concentrations of the constituents used in the simulation of each system are provided for each formulated model. The simulation process employs the law of mass conservation of intermediates as a prerequisite.

(i) The set of rate constants associated with each of the systems was evaluated by an optimized fit curve overlaid on the experimental results.

(ii) To support the derived set of rate constants as a meaningful solution for the system (rather than a coincidental local solution), we used the set of rate constants to predict the behavior of the system under different auxiliary conditions, and verified the predicted results through experiments.

(iii) We searched to support the computationally simulated results by identifying one of the sub-reactions that could be independently characterized experimentally. Specifically, for the systems described in the present study, the sub-reaction (2) that is common for all reaction schemes was experimentally validated. The reaction module validating the constant is described in Figure S11, where fluorophore/quencher-modified strands were used to model the sub-reaction (2), where $k_2 \approx k_{11}$ and $k_{-2} \approx k_{-11}$. (Details of this experiment are provided in Figure S11. We found that the computationally evaluated rate constants correspond to k_2 (k_{11}) = 18.6 μM^{-1} min^{-1} and k_{-2} (k_{-11}) = 1.0×10^{-6} $min^{-1,}$ and the experimental rate constants of the analog sub-reaction correspond to k_{11} = 18.5 μM^{-1} min^{-1} and k_{-11} = 1.0×10^{-6} min^{-1}, indicating that the computational rate constants are well supported by the experimental results.

(iv) As the kinetic models for all systems include a common set of rate constants with the need to add appropriate sub-reactions to account for the enhanced complexity of the respective systems, the simulated rate constants underwent sequential optimization for all of the systems, so that the entire set of rate constant represents a global set of consistent rate constants that account for all relevant systems in the study. The tables of rate constants associated with each of the systems discussed in the study are summarized in this supplemental information.

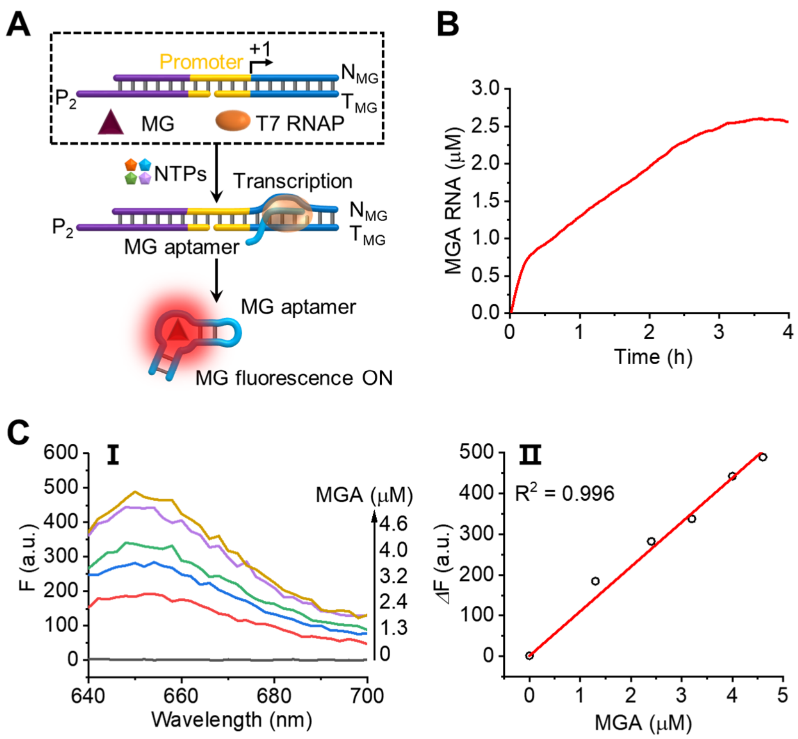

Figure S6. (A) Schematic illustration of the transcription-guided synthesis of MG aptamer, which binds to MG and "light up" MG fluorescence. (B) The temporal concentration of the transcribed RNA—MG aptamer by using the appropriate calibration curve shown in (C). The reaction module consists of $N_{MG}/T_{MG}+P_2 = 0.2\ \mu M$, NTPs = 0.5 mM), T7 RNAP = 2 U/μL, MG = 5 μM. (C) Panel I: The fluorescence spectra of MG (λ_{ex} = 632 nm) upon the addition of variable standard concentrations of MG aptamer. Panel II: The derived calibration curves corresponding to the fluorescence changes of MG (λ_{em} = 650 nm) with different concentrations of MG aptamer.

Calculation of RNA yield (*Y*)

The RNA yield (*Y*) was calculated according to equation (i):

$$Y = n_{RNA}/(n_{NTP}/N_{max}) \times 100\% = C_{RNA}/(C_{NTP}/N_{max}) \times 100\% \qquad \text{(i)}$$

As shown in Figure S6B, the final concentration of the transcribed RNA was 2.5 μM. The input concentration of NTPs fuel was 500 μM. Since the transcribed RNA contains 14 guanine residues (N_{max} = 14), this gives

$$Y = 2.5\ \mu M/(500\ \mu M/14) \times 100\% = 7\%$$

The RNA yield (*Y*) of 7% was then applied to all the kinetic models for the respective computational simulations.

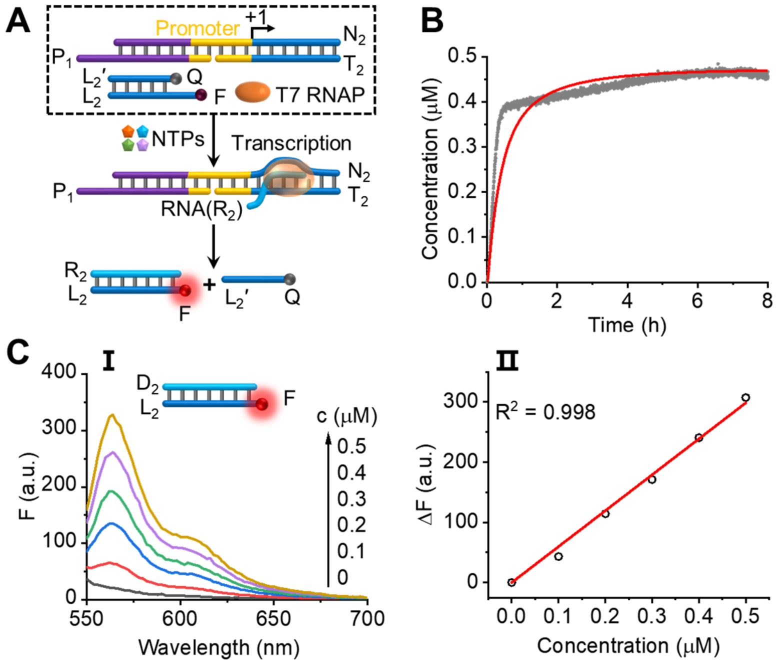

Figure S7. (A) Schematic illustration of real-time fluorescence monitoring of the transcription machinery. The addition of NTPs triggers the transcription of RNA (R_2), which displaces the Cy3-labeled L_2/BHQ2-labeled L_2' to form a Cy3-labeled L_2/R_2 duplex and BHQ2-labeled L_2', resulting in recovery of Cy3 fluorescence. (B) The temporal concentration of the displaced Cy3-labeled L_2 during the transcription. Dots correspond to the experimental data derived by using the calibration curve shown in (C). Solid curve corresponds to the computationally fitted concentration using the kinetic model presented in Figure S8 (the rate constants are summarized in Table S2). The reaction module consists of N_2/T_2+P_1 = 0.2 μM, Cy3-labeled L_2/BHQ2-labeled L_2' = 0.5 μM, T7 RNAP = 3 U/μL (0.048 μM), and NTPs = 0.5 mM. (C) Panel I: The fluorescence spectra of the displaced Cy3-labeled L_2 (λ_{ex} = 540 nm) upon addition of variable concentrations of D_2 to displace Cy3-labeled L_2/BHQ2-labeled L_2'. Panel II: The derived calibration curves corresponding to the fluorescence changes (λ_{em} = 564 nm) at different concentrations of displaced Cy3-labeled L_2.

Kinetic equations for the real-time fluorescence monitoring of the transcription:

(10) $N_2T_2 + P_1 \underset{k_{-10}}{\overset{k_{10}}{\rightleftarrows}} N_2T_2P_1$ (Activation)

(11) $L_2 + L_2' \underset{k_{-11}}{\overset{k_{11}}{\rightleftarrows}} L_2L_2'$

(12) $N_2T_2P_1 + \text{RNAP} \underset{k_{-12}}{\overset{k_{12}}{\rightleftarrows}} N_2T_2P_1\bullet\text{RNAP}$

(13) $N_2T_2P_1\bullet\text{RNAP} + \text{NTPs} \xrightarrow{k_{13}} \text{RNAP} + N_2T_2P_1 + R_2$ (length = 25, N_{max} (G) = 8)

(14) $R_2 + L_2L_2' \underset{k_{-14}}{\overset{k_{14}}{\rightleftarrows}} R_2L_2 + L_2'$

Derivatives:

$$\frac{dN_2T_2}{dt} = k_{-10}[N_2T_2P_1] - k_{10}[N_2T_2][P_1]$$

$$\frac{dP_1}{dt} = k_{-10}[N_2T_2P_1] - k_{10}[N_2T_2][P_1]$$

$$\frac{dN_2T_2P_1}{dt} = k_{10}[N_2T_2][P_1] - k_{-10}[N_2T_2P_1] - k_{12}[N_2T_2P_1][\text{RNAP}] + k_{-12}[N_2T_2P_1\bullet\text{RNAP}] + k_{13}[N_2T_2P_1\bullet\text{RNAP}][\text{NTPs}]\times 0.07/8$$

$$\frac{dL_2}{dt} = k_{-11}[L_2L_2'] - k_{11}[L_2][L_2']$$

$$\frac{dL_2'}{dt} = k_{-11}[L_2L_2'] - k_{11}[L_2][L_2'] + k_{14}[R_2][L_2L_2'] - k_{-14}[R_2L_2][L_2']$$

$$\frac{dL_2L_2'}{dt} = k_{11}[L_2][L_2'] - k_{-11}[L_2L_2'] - k_{14}[R_2][L_2L_2'] + k_{-14}[R_2L_2][L_2']$$

$$\frac{d\text{RNAP}}{dt} = k_{-12}[N_2T_2P_1\bullet\text{RNAP}] - k_{12}[N_2T_2P_1][\text{RNAP}] + k_{13}[N_2T_2P_1\bullet\text{RNAP}][\text{NTPs}]\times 0.07/8$$

$$\frac{dN_2T_2P_1\bullet\text{RNAP}}{dt} = k_{12}[N_2T_2P_1][\text{RNAP}] - k_{-12}[N_2T_2P_1\bullet\text{RNAP}] - k_{13}[N_2T_2P_1\bullet\text{RNAP}][\text{NTPs}]\times 0.07/8$$

$$\frac{d\text{NTPs}}{dt} = -k_{13}[N_2T_2P_1\bullet\text{RNAP}][\text{NTPs}]$$

$$\frac{dR_2}{dt} = k_{13}[N_2T_2P_1\bullet\text{RNAP}][\text{NTPs}]\times 0.07/8 - k_{14}[R_2][L_2L_2'] + k_{-14}[R_2L_2][L_2']$$

$$\frac{dR_2L_2}{dt} = k_{14}[R_2][L_2L_2'] - k_{-14}[R_2L_2][L_2']$$

Figure S8. Computational simulation of the real-time fluorescence monitoring of the transcription machinery shown in Figure S7. The kinetic scheme of the sub-reactions associated with the time-dependent concentration changes during the dissipative transitions are summarized in the above equations. Knowing the time-dependent concentration changes of the displaced Cy3-labeled L_2 (R_2L_2) during transcription, we computationally simulated the system by using Matlab R2019b. Initial concentrations of the condition: N_2/T_2+P_1 = 0.2 μM, Cy3-labeled L_2/BHQ2-labeled L_2' = 0.5 μM, T7 RNAP = 0.048 μM, and NTPs = 0.5 mM. The derived rate constants are summarized in Table S2.

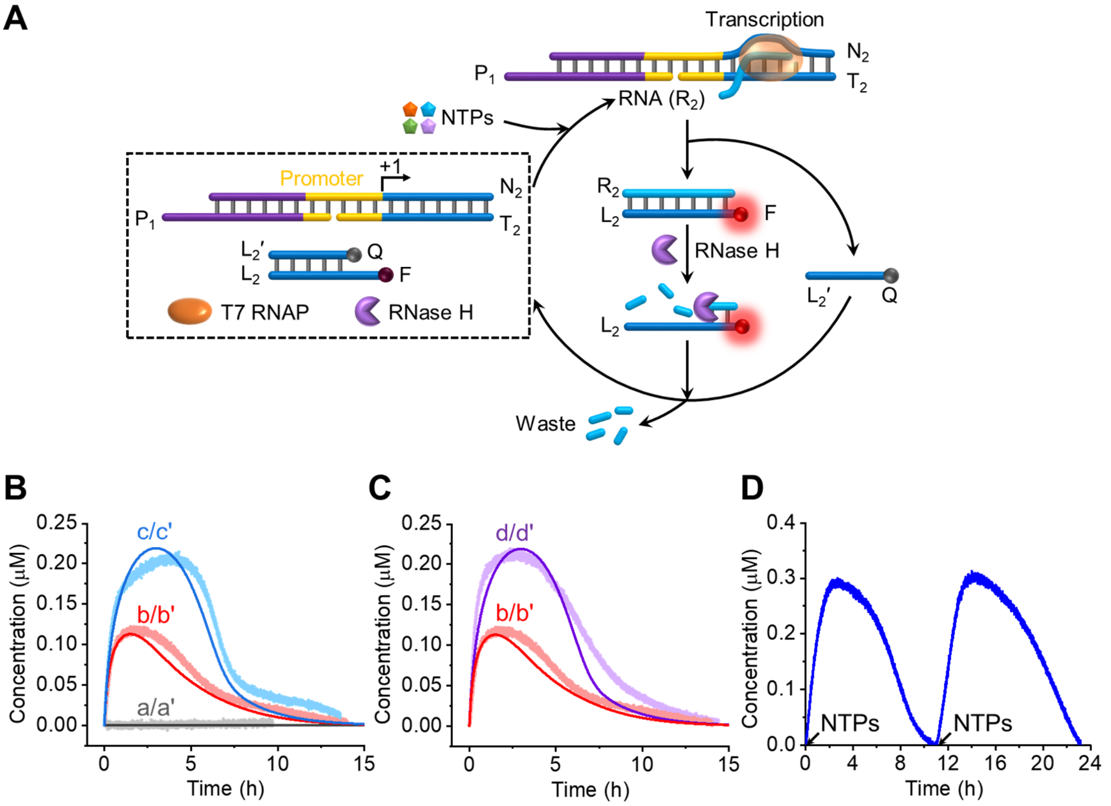

Figure S9. (A) Schematic illustration of real-time fluorescence monitoring of transcription machinery-guided transient synthesis and dissipative degradation of RNA. The addition of NTPs triggers the transcription of RNA (R_2), which displaces the Cy3-labeled L_2/BHQ2-labeled L_2' to form Cy3-labeled L_2/R_2 duplex and the BHQ2-labeled L_2', leading to the recovery of Cy3 fluorescence. The resulting Cy3-labeled L_2/R_2 duplex is, however, cleaved by RNase H to release Cy3-labeled L_2 that binds to BHQ2-labeled L_2' to regenerate the rest reaction module. (B) Temporal concentrations of the displaced Cy3-labeled L_2 in the presence of different concentrations of NTPs fuel: (a/a') 0 mM, (b/b') 0.5 mM, (c/c') 1 mM. Dotted curves a, b and c are experimental results. Solid lines a', b', and c' are computationally fitted results using the kinetic model in Figure S10. The set of rate constants (summarized in Table S3) are derived from curve b and then applied to predict the behavior of the network in the presence of NTPs fuel: (a') 0 mM and (c') 1 mM. The network module consists of N_2/T_2+P_1 = 0.2 μM, Cy3-labeled L_2/BHQ2-labeled L_2' = 0.5 μM, T7 RNAP = 2 U/μL (0.032 μM), and RNase H = 8 U/mL (0.266 nM). (C) Temporal concentrations of the displaced Cy3-labeled L_2 in the presence of NTPs, 0.5 mM, and different concentrations of RNase H: (b/b') 8 U/mL, (d/d') 6 U/mL (0.199 nM). All other conditions are the same as those described in (B). Solid line d' is computationally predicted result, and dotted curve d is experimentally validated result. (D) Operation of two dissipative cycles upon re-addition of NTP fuels, 0.5 mM, after the first cycle. The network module consists of N_2/T_2+P_1 = 0.2 μM, Cy3-labeled L_2/BHQ2-labeled L_2' = 0.5 μM, T7 RNAP = 2 U/μL (0.032 μM), and RNase H = 5 U/mL (0.166 nM).

Kinetic equations for real-time fluorescence monitoring of transcription machinery-guided dissipative synthesis and degradation of RNA:

(10) $N_2T_2 + P_1 \underset{k_{-10}}{\overset{k_{10}}{\rightleftarrows}} N_2T_2P_1$ (Activation)

(11) $L_2 + L_2' \underset{k_{-11}}{\overset{k_{11}}{\rightleftarrows}} L_2L_2'$

(12) $N_2T_2P_1 + \text{RNAP} \underset{k_{-12}}{\overset{k_{12}}{\rightleftarrows}} N_2T_2P_1{\bullet}\text{RNAP}$

(13) $N_2T_2P_1{\bullet}\text{RNAP} + \text{NTPs} \xrightarrow{k_{13}} \text{RNAP} + N_2T_2P_1 + R_2$ (length = 25, $N_{\max}$ (G) = 8)

(14) $R_2 + L_2L_2' \underset{k_{-14}}{\overset{k_{14}}{\rightleftarrows}} R_2L_2 + L_2'$

(15) $R_2L_2 + \text{RNaseH} \underset{k_{-15}}{\overset{k_{15}}{\rightleftarrows}} R_2L_2{\bullet}\text{RNaseH}$

(16) $R_2L_2{\bullet}\text{RNaseH} \xrightarrow{k_{16}} \text{RNaseH} + L_2Waste_2$

(17) $L_2Waste_2 \underset{k_{-17}}{\overset{k_{17}}{\rightleftarrows}} L_2 + Waste_2$

Derivatives:

$$\frac{dN_2T_2}{dt} = k_{-10}[N_2T_2P_1] - k_{10}[N_2T_2][P_1]$$

$$\frac{dP_1}{dt} = k_{-10}[N_2T_2P_1] - k_{10}[N_2T_2][P_1]$$

$$\frac{dN_2T_2P_1}{dt} = k_{10}[N_2T_2][P_1] - k_{-10}[N_2T_2P_1] - k_{12}[N_2T_2P_1][\text{RNAP}] + k_{-12}[N_2T_2P_1{\bullet}\text{RNAP}] + k_{13}[N_2T_2P_1{\bullet}\text{RNAP}][\text{NTPs}] \times 0.07/8$$

$$\frac{dL_2}{dt} = k_{-11}[L_2L_2'] - k_{11}[L_2][L_2'] + k_{17}[L_2Waste_2] - k_{-17}[L_2][Waste_2]$$

$$\frac{dL_2'}{dt} = k_{-11}[L_2L_2'] - k_{11}[L_2][L_2'] + k_{14}[R_2][L_2L_2'] - k_{-14}[R_2L_2][L_2']$$

$$\frac{dL_2L_2'}{dt} = k_{11}[L_2][L_2'] - k_{-11}[L_2L_2'] - k_{14}[R_2][L_2L_2'] + k_{-14}[R_2L_2][L_2']$$

$$\frac{d\text{RNAP}}{dt} = k_{-12}[N_2T_2P_1{\bullet}\text{RNAP}] - k_{12}[N_2T_2P_1][\text{RNAP}] + k_{13}[N_2T_2P_1{\bullet}\text{RNAP}][\text{NTPs}] \times 0.07/8$$

$$\frac{dN_2T_2P_1{\bullet}\text{RNAP}}{dt} = k_{12}[N_2T_2P_1][\text{RNAP}] - k_{-12}[N_2T_2P_1{\bullet}\text{RNAP}] - k_{13}[N_2T_2P_1{\bullet}\text{RNAP}][\text{NTPs}] \times 0.07/8$$

$$\frac{d\text{NTPs}}{dt} = -k_{13}[N_2T_2P_1{\bullet}\text{RNAP}][\text{NTPs}]$$

$$\frac{dR_2}{dt} = k_{13}[N_2T_2P_1{\bullet}\text{RNAP}][\text{NTPs}] \times 0.07/8 - k_{14}[R_2][L_2L_2'] + k_{-14}[R_2L_2][L_2']$$

$$\frac{dR_2L_2}{dt} = k_{14}[R_2][L_2L_2'] - k_{-14}[R_2L_2][L_2'] - k_{15}[R_2L_2][\text{RNaseH}] + k_{-15}[R_2L_2{\bullet}\text{RNaseH}]$$

$$\frac{d\text{RNaseH}}{dt} = k_{-15}[R_2L_2{\bullet}\text{RNaseH}] - k_{15}[R_2L_2][\text{RNaseH}] + k_{16}[R_2L_2{\bullet}\text{RNaseH}]$$

$$\frac{dR_2L_2{\bullet}\text{RNaseH}}{dt} = k_{15}[R_2L_2][\text{RNaseH}] - k_{-15}[R_2L_2{\bullet}\text{RNaseH}] - k_{16}[R_2L_2{\bullet}\text{RNaseH}]$$

$$\frac{dL_2Waste_2}{dt} = k_{16}[R_2L_2{\bullet}\text{RNaseH}] - k_{17}[L_2Waste_2] + k_{-17}[L_2][Waste_2]$$

$$\frac{dWaste_2}{dt} = k_{17}[L_2Waste_2] - k_{-17}[L_2][Waste_2]$$

Figure S10. Computational simulation of the real-time fluorescence monitoring of the transient transcription machinery-guided dissipative synthesis and degradation of RNA shown in Figure S9. The kinetic scheme of the reactions associated with the time-dependent concentration changes during the dissipative transitions is summarized in the above equations. Knowing the time-dependent concentration changes of the displaced Cy3-labeled L_2 (R_2L_2), during the dissipative transitions, we computationally simulated the system by using Matlab R2019b. Initial concentrations of the condition: N_2/T_2+P_1 = 0.2 μM, Cy3-labeled L_2/BHQ2-labeled L_2' = 0.5 μM, T7 RNAP = 0.032 μM, and RNase H = 0.266 nM, NTPs = 0.5 mM. The derived rate constants are summarized in Table S3.

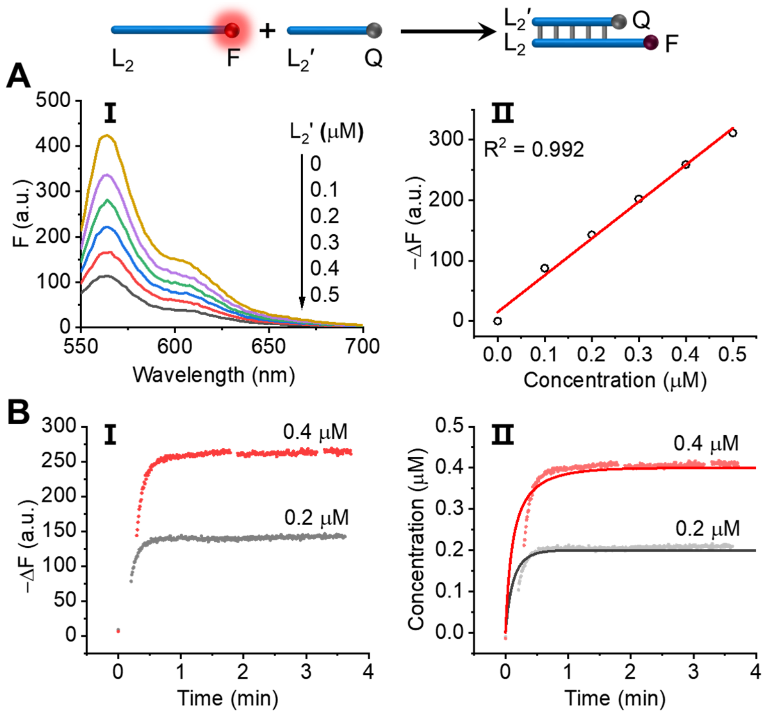

Figure S11. (A) Panel I: The fluorescence spectra of Cy3-labeled L_2 (0.5 μM) upon addition of variable concentrations of BHQ2-labeled L_2'. Panel II: The derived calibration curve corresponding to the decrease in fluorescence intensity (λ_{em} = 564 nm) at different concentrations of BHQ2-labeled L_2'. (B) Time-dependent fluorescence changes (Panel I) and time-dependent concentration changes of Cy3-labeled L_2/ BHQ2-labeled L_2' (Panel II) upon subjecting the Cy3-labeled L_2 (0.5 μM) to BHQ2-labeled L_2': 0.2 μM (gray) and 0.4 μM (red). Dots correspond to the experimental data and solid curves correspond to the computationally simulated kinetic profiles.

Experimental validation of the rate constants k_{11} and k_{-11}

To experimentally evaluate the rate constants k_{11} and k_{-11} appearing in the kinetic scheme depicted in Figures S8 and S10, we subjected the Cy3-labeled L_2 (0.5 μM) to two concentrations of BHQ2-labeled L_2' (0.2 μM and 0.4 μM). The time-dependent concentration changes of Cy3-labeled L_2/ BHQ2-labeled L_2' were evaluated by following the time-dependent fluorescence changes of Cy3 at λ_{em} = 564 nm and translating them into concentrations by applying the appropriate calibration curve in Figure S11A. From the kinetic profiles and using the Matlab R2019b program, the respective k_{11} = 18.5 μM^{-1} min^{-1} and k_{-11} = 1.0×10^{-6} min^{-1} were derived. The experimental results well support the computationally evaluated rate constants correspond to k_2 (k_{11}) = 18.6 μM^{-1} min^{-1} and k_{-2} (k_{-11}) = 1.0×10^{-6} min^{-1}.

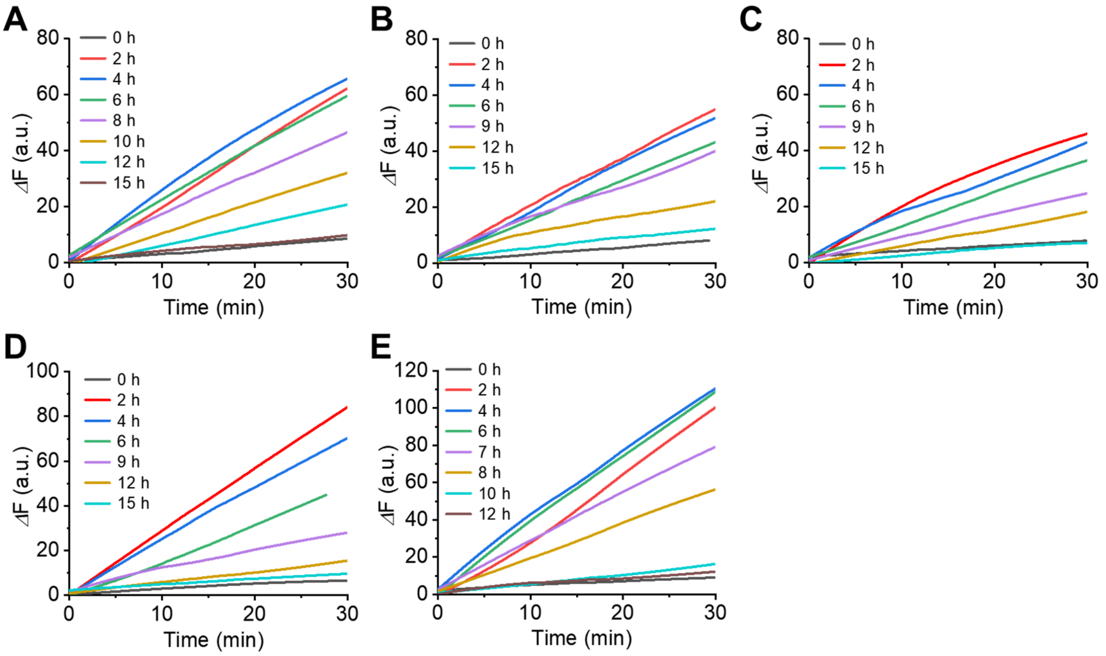

Figure S12. Temporal, time-dependent fluorescence changes generated by the intermediate DNAzyme α, upon catalyzed cleavage of its fluorophore/quencher-modified substrate S_1, formed at different time intervals of transient generation and depletion according to Figure 1A, under different auxiliary conditions: (A) NTPs = 1 mM, T7 RNAP = 2 U/μL (0.032 μM), and RNase H = 6 U/mL (0.199 nM); (B) NTPs = 1 mM, T7 RNAP = 2 U/μL (0.032 μM), and RNase H = 7 U/mL (0.233 nM); (C) NTPs = 1 mM, T7 RNAP = 2 U/μL (0.032 μM), and RNase H = 8 U/mL (0.266 nM); (D) NTPs = 1 mM, T7 RNAP = 3 U/μL (0.048 μM), and RNase H = 8 U/mL (0.266 nM); (E) NTPs = 1 mM, T7 RNAP = 4 U/μL (0.064 μM), and RNase H = 8 U/mL (0.266 nM). Other conditions are N_1/T_1+P_1 = 0.2 μM, M_1/L_1 = 0.5 μM, M_2 = 0.5 μM.

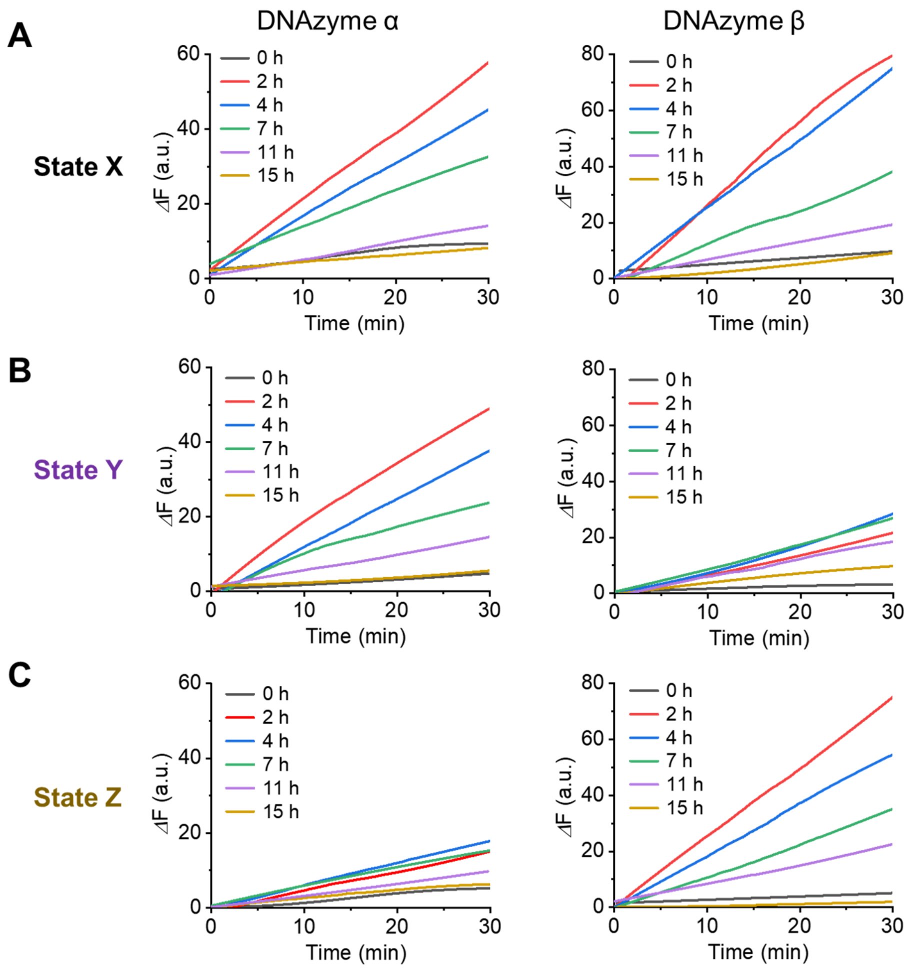

Figure S13. Time-dependent fluorescence changes generated from the cleavage of fluorophore/quencher-modified substrates S_1 or S_2 by the intermediate DNAzymes α or β, formed at different time intervals of the gated transient operation of two DNAzymes according to Figure 2: (A) State X, all inhibitors = 0 μM; (B) State Y, inhibitors I_{M2} = 0 μM, I_{M4} = 2 μM; (C) State Z, inhibitors I_{M2} = 2 μM, I_{M4} = 0 μM. For all gated systems: N_3/T_3 = 0.2 μM, M_1/L_1 = 0.5 μM, M_2 = 0.5 μM, M_3/L_3 = 0.5 μM, M_4 = 0.5 μM, T7 RNAP = 3 U/μL, RNase H = 8 U/mL, NTPs = 1 mM.

Kinetic equations of the transcription machinery-guided, parallel, non-gated operation of two dissipative DNAzymes (state X) shown in Figure 2:

(18) $N_3 + T_3 \underset{k_{-18}}{\overset{k_{18}}{\rightleftarrows}} N_3T_3$

(2) $M_1 + L_1 \underset{k_{-2}}{\overset{k_{2}}{\rightleftarrows}} M_1L_1$

(19) $M_3 + L_3 \underset{k_{-19}}{\overset{k_{19}}{\rightleftarrows}} M_3L_3$

(20) $N_3T_3 + \text{RNAP} \underset{k_{-20}}{\overset{k_{20}}{\rightleftarrows}} N_3T_3\bullet\text{RNAP}$

(21) $N_3T_3\bullet\text{RNAP} + \text{NTPs} \xrightarrow{k_{21}} \text{RNAP} + N_3T_3 + R_3$ (length = 42, $N_{\max}$ (G) = 14)

(22) $R_3 + M_1L_1 + M_3L_3 \underset{k_{-22}}{\overset{k_{22}}{\rightleftarrows}} R_3L_1L_3 + M_1 + M_3$

(6) $M_1 + M_2 \underset{k_{-6}}{\overset{k_{6}}{\rightleftarrows}} M_1M_2$

(23) $M_3 + M_4 \underset{k_{-23}}{\overset{k_{23}}{\rightleftarrows}} M_3M_4$

(24) $R_3L_1L_3 + \text{RNaseH} \underset{k_{-24}}{\overset{k_{24}}{\rightleftarrows}} R_3L_1L_3\bullet\text{RNaseH}$

(25) $R_3L_1L_3\bullet\text{RNaseH} \xrightarrow{k_{25}} \text{RNaseH} + L_1L_3Waste_3$

(26) $L_1L_3Waste_3 \underset{k_{-26}}{\overset{k_{26}}{\rightleftarrows}} L_1 + L_3 + Waste_3$

Derivatives:

$$\frac{dN_3}{dt} = k_{-18}[N_3T_3] - k_{18}[N_3][T_3]$$

$$\frac{dT_3}{dt} = k_{-18}[N_3T_3] - k_{18}[N_3][T_3]$$

$$\frac{dN_3T_3}{dt} = k_{18}[N_3][T_3] - k_{-18}[N_3T_3] - k_{20}[N_3T_3][\text{RNAP}] + k_{-20}[N_3T_3\bullet\text{RNAP}]$$
$$+ k_{21}[N_3T_3\bullet\text{RNAP}][\text{NTPs}] \times 0.07/14$$

$$\frac{dM_1}{dt} = k_{-2}[M_1L_1] - k_2[M_1][L_1] + k_{-6}[M_1M_2] - k_6[M_1][M_2] + k_{22}[R_3][M_1L_1][M_3L_3]$$
$$- k_{-22}[R_3L_1L_3][M_1][M_3]$$

$$\frac{dL_1}{dt} = k_{-2}[M_1L_1] - k_2[M_1][L_1] + k_{26}[L_1L_3Waste_3] - k_{-26}[L_1][L_3][Waste_3]$$

$$\frac{dM_1L_1}{dt} = k_2[M_1][L_1] - k_{-2}[M_1L_1] - k_{22}[R_3][M_1L_1][M_3L_3] + k_{-22}[R_3L_1L_3][M_1][M_3]$$

$$\frac{dM_3}{dt} = k_{-19}[M_3L_3] - k_{19}[M_3][L_3] + k_{-23}[M_3M_4] - k_{23}[M_3][M_4] + k_{22}[R_3][M_1L_1][M_3L_3]$$
$$- k_{-22}[R_3L_1L_3][M_1][M_3]$$

$$\frac{dL_3}{dt} = k_{-19}[M_3L_3] - k_{19}[M_3][L_3] + k_{26}[L_1L_3Waste_3] - k_{-26}[L_1][L_3][Waste_3]$$

$$\frac{dM_3L_3}{dt} = k_{19}[M_3][L_3] - k_{-19}[M_3L_3] - k_{22}[R_3][M_1L_1][M_3L_3] + k_{-22}[R_3L_1L_3][M_1][M_3]$$

$$\frac{d\text{RNAP}}{dt} = k_{-20}[N_3T_3\bullet\text{RNAP}] - k_{20}[N_3T_3][\text{RNAP}] + k_{21}[N_3T_3\bullet\text{RNAP}][\text{NTPs}] \times 0.07/14$$

$$\frac{dN_3T_3\bullet\text{RNAP}}{dt} = k_{20}[N_3T_3][\text{RNAP}] - k_{-20}[N_3T_3\bullet\text{RNAP}] - k_{21}[N_3T_3\bullet\text{RNAP}][\text{NTPs}] \times 0.07/14$$

$$\frac{d\text{NTPs}}{dt} = -k_{21}[N_3T_3\bullet\text{RNAP}][\text{NTPs}]$$

$$\frac{dR_3}{dt} = k_{21}[N_3T_3\bullet\text{RNAP}][\text{NTPs}] \times 0.07/14 - k_{22}[R_3][M_1L_1][M_3L_3] + k_{-22}[R_3L_1L_3][M_1][M_3]$$

$$\frac{dR_3L_1L_3}{dt} = k_{22}[R_3][M_1L_1][M_3L_3] - k_{-22}[R_3L_1L_3][M_1][M_3] - k_{24}[R_3L_1L_3][\text{RNaseH}] + k_{-24}[R_3L_1L_3\bullet\text{RNaseH}]$$

$$\frac{dM_2}{dt} = k_{-6}[M_1M_2] - k_6[M_1][M_2]$$

$$\frac{dM_1M_2}{dt} = k_6[M_1][M_2] - k_{-6}[M_1M_2]$$

$$\frac{dM_4}{dt} = k_{-23}[M_3M_4] - k_{23}[M_3][M_4]$$

$$\frac{dM_3M_4}{dt} = k_{23}[M_3][M_4] - k_{-23}[M_3M_4]$$

$$\frac{d\text{RNaseH}}{dt} = k_{-24}[R_3L_1L_3\bullet\text{RNaseH}] - k_{24}[R_3L_1L_3][\text{RNaseH}] + k_{25}[R_3L_1L_3\bullet\text{RNaseH}]$$

$$\frac{dR_3L_1L_3\bullet\text{RNaseH}}{dt} = k_{24}[R_3L_1L_3][\text{RNaseH}] - k_{-24}[R_3L_1L_3\bullet\text{RNaseH}] - k_{25}[R_3L_1L_3\bullet\text{RNaseH}]$$

$$\frac{dL_1L_3Waste_3}{dt} = k_{25}[R_3L_1L_3\bullet\text{RNaseH}] - k_{26}[L_1L_3Waste_3] + k_{-26}[L_1][L_3][Waste_3]$$

$$\frac{dWaste_3}{dt} = k_{26}[L_1L_3Waste_3] - k_{-26}[L_1][L_3][Waste_3]$$

Figure S14. Computational simulation of the transcription machinery-guided, parallel, non-gated operation of two dissipative DNAzymes (state X) shown in Figure 2. The kinetic scheme of the sub-reactions associated with the temporal concentration changes during the dissipative transitions is summarized in the above equations. Knowing the time-dependent concentration changes of M_1/M_2 and M_3/M_4 during the dissipative transitions, we computationally simulated the time-dependent concentration changes by using Matlab R2019b. Initial concentrations of the condition: N_3/T_3 = 0.2 μM, M_1/L_1 = 0.5 μM, M_2 = 0.5 μM, M_3/L_3 = 0.5 μM, M_4 = 0.5 μM, NTPs = 1 mM, T7 RNAP = 0.048 μM, and RNase H = 0.266 nM. The derived rate constants are summarized in Table S4.

Kinetic equations of the inhibitor I_{M4}-gated transcription machinery-guided transient operation of two DNAzymes (state Y) shown in Figure 2:

(18) $N_3 + T_3 \underset{k_{-18}}{\overset{k_{18}}{\rightleftarrows}} N_3T_3$

(2) $M_1 + L_1 \underset{k_{-2}}{\overset{k_2}{\rightleftarrows}} M_1L_1$

(19) $M_3 + L_3 \underset{k_{-19}}{\overset{k_{19}}{\rightleftarrows}} M_3L_3$

(20) $N_3T_3 + \text{RNAP} \underset{k_{-20}}{\overset{k_{20}}{\rightleftarrows}} N_3T_3{\bullet}\text{RNAP}$

(21) $N_3T_3{\bullet}\text{RNAP} + \text{NTPs} \xrightarrow{k_{21}} \text{RNAP} + N_3T_3 + R_3$ (length = 42, N_{max} (G) = 14)

(22) $R_3 + M_1L_1 + M_3L_3 \underset{k_{-22}}{\overset{k_{22}}{\rightleftarrows}} R_3L_1L_3 + M_1 + M_3$

(6) $M_1 + M_2 \underset{k_{-6}}{\overset{k_6}{\rightleftarrows}} M_1M_2$

(27) $I_{M4} + M_4 \underset{k_{-27}}{\overset{k_{27}}{\rightleftarrows}} I_{M4}M_4$

(28) $M_3 + I_{M4}M_4 \underset{k_{-28}}{\overset{k_{28}}{\rightleftarrows}} M_3M_4 + I_{M4}$

(24) $R_3L_1L_3 + \text{RNaseH} \underset{k_{-24}}{\overset{k_{24}}{\rightleftarrows}} R_3L_1L_3{\bullet}\text{RNaseH}$

(25) $R_3L_1L_3{\bullet}\text{RNaseH} \xrightarrow{k_{25}} \text{RNaseH} + L_1L_3Waste_3$

(26) $L_1L_3Waste_3 \underset{k_{-26}}{\overset{k_{26}}{\rightleftarrows}} L_1 + L_3 + Waste_3$

Derivatives:

$$\frac{dN_3}{dt} = k_{-18}[N_3T_3] - k_{18}[N_3][T_3]$$

$$\frac{dT_3}{dt} = k_{-18}[N_3T_3] - k_{18}[N_3][T_3]$$

$$\frac{dN_3T_3}{dt} = k_{18}[N_3][T_3] - k_{-18}[N_3T_3] - k_{20}[N_3T_3][\text{RNAP}] + k_{-20}[N_3T_3{\bullet}\text{RNAP}]$$
$$+ k_{21}[N_3T_3{\bullet}\text{RNAP}][\text{NTPs}] \times 0.07/14$$

$$\frac{dM_1}{dt} = k_{-2}[M_1L_1] - k_2[M_1][L_1] + k_{-6}[M_1M_2] - k_6[M_1][M_2] + k_{22}[R_3][M_1L_1][M_3L_3]$$
$$- k_{-22}[R_3L_1L_3][M_1][M_3]$$

$$\frac{dL_1}{dt} = k_{-2}[M_1L_1] - k_2[M_1][L_1] + k_{26}[L_1L_3Waste_3] - k_{-26}[L_1][L_3][Waste_3]$$

$$\frac{dM_1L_1}{dt} = k_2[M_1][L_1] - k_{-2}[M_1L_1] - k_{22}[R_3][M_1L_1][M_3L_3] + k_{-22}[R_3L_1L_3][M_1][M_3]$$

$$\frac{dM_3}{dt} = k_{-19}[M_3L_3] - k_{19}[M_3][L_3] + k_{22}[R_3][M_1L_1][M_3L_3] - k_{-22}[R_3L_1L_3][M_1][M_3]$$
$$- k_{28}[M_3][I_{M4}M_4] + k_{-28}[I_{M4}][M_3M_4]$$

$$\frac{dL_3}{dt} = k_{-19}[M_3L_3] - k_{19}[M_3][L_3] + k_{26}[L_1L_3Waste_3] - k_{-26}[L_1][L_3][Waste_3]$$

$$\frac{dM_3L_3}{dt} = k_{19}[M_3][L_3] - k_{-19}[M_3L_3] - k_{22}[R_3][M_1L_1][M_3L_3] + k_{-22}[R_3L_1L_3][M_1][M_3]$$

$$\frac{d\text{RNAP}}{dt} = k_{-20}[N_3T_3\bullet\text{RNAP}] - k_{20}[N_3T_3][\text{RNAP}] + k_{21}[N_3T_3\bullet\text{RNAP}][\text{NTPs}]\times 0.07/14$$

$$\frac{dN_3T_3\bullet\text{RNAP}}{dt} = k_{20}[N_3T_3][\text{RNAP}] - k_{-20}[N_3T_3\bullet\text{RNAP}] - k_{21}[N_3T_3\bullet\text{RNAP}][\text{NTPs}]\times 0.07/14$$

$$\frac{d\text{NTPs}}{dt} = -k_{21}[N_3T_3\bullet\text{RNAP}][\text{NTPs}]$$

$$\frac{dR_3}{dt} = k_{21}[N_3T_3\bullet\text{RNAP}][\text{NTPs}]\times 0.07/14 - k_{22}[R_3][M_1L_1][M_3L_3] + k_{-22}[R_3L_1L_3][M_1][M_3]$$

$$\frac{dR_3L_1L_3}{dt} = k_{22}[R_3][M_1L_1][M_3L_3] - k_{-22}[R_3L_1L_3][M_1][M_3] - k_{24}[R_3L_1L_3][\text{RNaseH}] + k_{-24}[R_3L_1L_3\bullet\text{RNaseH}]$$

$$\frac{dM_2}{dt} = k_{-6}[M_1M_2] - k_6[M_1][M_2]$$

$$\frac{dM_1M_2}{dt} = k_6[M_1][M_2] - k_{-6}[M_1M_2]$$

$$\frac{dI_{M4}}{dt} = k_{-27}[I_{M4}M_4] - k_{27}[I_{M4}][M_4] + k_{28}[M_3][I_{M4}M_4] - k_{-28}[I_{M4}][M_3M_4]$$

$$\frac{dM_4}{dt} = k_{-27}[I_{M4}M_4] - k_{27}[I_{M4}][M_4]$$

$$\frac{dI_{M4}M_4}{dt} = k_{27}[I_{M4}][M_4] - k_{-27}[I_{M4}M_4] - k_{28}[M_3][I_{M4}M_4] + k_{-28}[I_{M4}][M_3M_4]$$

$$\frac{dM_3M_4}{dt} = k_{28}[M_3][I_{M4}M_4] - k_{-28}[I_{M4}][M_3M_4]$$

$$\frac{d\text{RNaseH}}{dt} = k_{-24}[R_3L_1L_3\bullet\text{RNaseH}] - k_{24}[R_3L_1L_3][\text{RNaseH}] + k_{25}[R_3L_1L_3\bullet\text{RNaseH}]$$

$$\frac{dR_3L_1L_3\bullet\text{RNaseH}}{dt} = k_{24}[R_3L_1L_3][\text{RNaseH}] - k_{-24}[R_3L_1L_3\bullet\text{RNaseH}] - k_{25}[R_3L_1L_3\bullet\text{RNaseH}]$$

$$\frac{dL_1L_3Waste_3}{dt} = k_{25}[R_3L_1L_3\bullet\text{RNaseH}] - k_{26}[L_1L_3Waste_3] + k_{-26}[L_1][L_3][Waste_3]$$

$$\frac{dWaste_3}{dt} = k_{26}[L_1L_3Waste_3] - k_{-26}[L_1][L_3][Waste_3]$$

Figure S15. Computational simulation of the inhibitor I_{M4}-gated transcription machinery-guided transient operation of two DNAzymes (state Y) shown in Figure 2. The kinetic scheme of the sub-reactions associated with the time-dependent concentration changes during the dissipative transitions is summarized in the above equations. Knowing the time-dependent concentration changes of M_1/M_2 and M_3/M_4 during the dissipative transitions, we computationally simulated the time-dependent concentration changes by using Matlab R2019b. Initial concentrations of the condition: N_3/T_3 = 0.2 μM, M_1/L_1 = 0.5 μM, M_2 = 0.5 μM, M_3/L_3 = 0.5 μM, M_4 = 0.5 μM, I_{M4} = 2 μM, NTPs = 1 mM, T7 RNAP = 0.048 μM, and RNase H = 0.266 nM. The derived rate constants are summarized in Table S5.

Kinetic equations of the inhibitor I_{M2}-gated transcription machinery-guided transient operation of two DNAzymes (state Z) shown in Figure 2:

(18) $N_3 + T_3 \underset{k_{-18}}{\overset{k_{18}}{\rightleftarrows}} N_3T_3$

(2) $M_1 + L_1 \underset{k_{-2}}{\overset{k_2}{\rightleftarrows}} M_1L_1$

(19) $M_3 + L_3 \underset{k_{-19}}{\overset{k_{19}}{\rightleftarrows}} M_3L_3$

(20) $N_3T_3 + \mathrm{RNAP} \underset{k_{-20}}{\overset{k_{20}}{\rightleftarrows}} N_3T_3\bullet\mathrm{RNAP}$

(21) $N_3T_3\bullet\mathrm{RNAP} + \mathrm{NTPs} \xrightarrow{k_{21}} \mathrm{RNAP} + N_3T_3 + R_3$ (length = 42, $N_{\max}$ (G) = 14)

(22) $R_3 + M_1L_1 + M_3L_3 \underset{k_{-22}}{\overset{k_{22}}{\rightleftarrows}} R_3L_1L_3 + M_1 + M_3$

(29) $I_{M2} + M_2 \underset{k_{-29}}{\overset{k_{29}}{\rightleftarrows}} I_{M2}M_2$

(30) $M_1 + I_{M2}M_2 \underset{k_{-30}}{\overset{k_{30}}{\rightleftarrows}} M_1M_2 + I_{M2}$

(23) $M_3 + M_4 \underset{k_{-23}}{\overset{k_{23}}{\rightleftarrows}} M_3M_4$

(24) $R_3L_1L_3 + \mathrm{RNaseH} \underset{k_{-24}}{\overset{k_{24}}{\rightleftarrows}} R_3L_1L_3\bullet\mathrm{RNaseH}$

(25) $R_3L_1L_3\bullet\mathrm{RNaseH} \xrightarrow{k_{25}} \mathrm{RNaseH} + L_1L_3Waste_3$

(26) $L_1L_3Waste_3 \underset{k_{-26}}{\overset{k_{26}}{\rightleftarrows}} L_1 + L_3 + Waste_3$

Derivatives:

$$\frac{dN_3}{dt} = k_{-18}[N_3T_3] - k_{18}[N_3][T_3]$$

$$\frac{dT_3}{dt} = k_{-18}[N_3T_3] - k_{18}[N_3][T_3]$$

$$\frac{dN_3T_3}{dt} = k_{18}[N_3][T_3] - k_{-18}[N_3T_3] - k_{20}[N_3T_3][\mathrm{RNAP}] + k_{-20}[N_3T_3\bullet\mathrm{RNAP}]$$
$$+ k_{21}[N_3T_3\bullet\mathrm{RNAP}][\mathrm{NTPs}] \times 0.07/14$$

$$\frac{dM_1}{dt} = k_{-2}[M_1L_1] - k_2[M_1][L_1] + k_{22}[R_3][M_1L_1][M_3L_3] - k_{-22}[R_3L_1L_3][M_1][M_3] - k_{30}[M_1][I_{M2}M_2]$$
$$+ k_{-30}[I_{M2}][M_1M_2]$$

$$\frac{dL_1}{dt} = k_{-2}[M_1L_1] - k_2[M_1][L_1] + k_{26}[L_1L_3Waste_3] - k_{-26}[L_1][L_3][Waste_3]$$

$$\frac{dM_1L_1}{dt} = k_2[M_1][L_1] - k_{-2}[M_1L_1] - k_{22}[R_3][M_1L_1][M_3L_3] + k_{-22}[R_3L_1L_3][M_1][M_3]$$

$$\frac{dM_3}{dt} = k_{-19}[M_3L_3] - k_{19}[M_3][L_3] + k_{-23}[M_3M_4] - k_{23}[M_3][M_4] + k_{22}[R_3][M_1L_1][M_3L_3]$$
$$- k_{-22}[R_3L_1L_3][M_1][M_3]$$

$$\frac{dL_3}{dt} = k_{-19}[M_3L_3] - k_{19}[M_3][L_3] + k_{26}[L_1L_3Waste_3] - k_{-26}[L_1][L_3][Waste_3]$$

$$\frac{dM_3L_3}{dt} = k_{19}[M_3][L_3] - k_{-19}[M_3L_3] - k_{22}[R_3][M_1L_1][M_3L_3] + k_{-22}[R_3L_1L_3][M_1][M_3]$$

$$\frac{d\mathrm{RNAP}}{dt} = k_{-20}[N_3T_3\bullet\mathrm{RNAP}] - k_{20}[N_3T_3][\mathrm{RNAP}] + k_{21}[N_3T_3\bullet\mathrm{RNAP}][\mathrm{NTPs}]\times 0.07/14$$

$$\frac{dN_3T_3\bullet\mathrm{RNAP}}{dt} = k_{20}[N_3T_3][\mathrm{RNAP}] - k_{-20}[N_3T_3\bullet\mathrm{RNAP}] - k_{21}[N_3T_3\bullet\mathrm{RNAP}][\mathrm{NTPs}]\times 0.07/14$$

$$\frac{d\mathrm{NTPs}}{dt} = -k_{21}[N_3T_3\bullet\mathrm{RNAP}][\mathrm{NTPs}]$$

$$\frac{dR_3}{dt} = k_{21}[N_3T_3\bullet\mathrm{RNAP}][\mathrm{NTPs}]\times 0.07/14 - k_{22}[R_3][M_1L_1][M_3L_3] + k_{-22}[R_3L_1L_3][M_1][M_3]$$

$$\frac{dR_3L_1L_3}{dt} = k_{22}[R_3][M_1L_1][M_3L_3] - k_{-22}[R_3L_1L_3][M_1][M_3] - k_{24}[R_3L_1L_3][\mathrm{RNaseH}] + k_{-24}[R_3L_1L_3\bullet\mathrm{RNaseH}]$$

$$\frac{dM_2}{dt} = k_{-29}[I_{M2}M_2] - k_{29}[I_{M2}][M_2]$$

$$\frac{dI_{M2}}{dt} = k_{-29}[I_{M2}M_2] - k_{29}[I_{M2}][M_2] + k_{30}[M_1][I_{M2}M_2] - k_{-30}[I_{M2}][M_1M_2]$$

$$\frac{dI_{M2}M_2}{dt} = k_{29}[I_{M2}][M_2] - k_{-29}[I_{M2}M_2] - k_{30}[M_1][I_{M2}M_2] + k_{-30}[I_{M2}][M_1M_2]$$

$$\frac{dM_1M_2}{dt} = k_{30}[M_1][I_{M2}M_2] - k_{-30}[I_{M2}][M_1M_2]$$

$$\frac{dM_4}{dt} = k_{-23}[M_3M_4] - k_{23}[M_3][M_4]$$

$$\frac{dM_3M_4}{dt} = k_{23}[M_3][M_4] - k_{-23}[M_3M_4]$$

$$\frac{d\mathrm{RNaseH}}{dt} = k_{-24}[R_3L_1L_3\bullet\mathrm{RNaseH}] - k_{24}[R_3L_1L_3][\mathrm{RNaseH}] + k_{25}[R_3L_1L_3\bullet\mathrm{RNaseH}]$$

$$\frac{dR_3L_1L_3\bullet\mathrm{RNaseH}}{dt} = k_{24}[R_3L_1L_3][\mathrm{RNaseH}] - k_{-24}[R_3L_1L_3\bullet\mathrm{RNaseH}] - k_{25}[R_3L_1L_3\bullet\mathrm{RNaseH}]$$

$$\frac{dL_1L_3Waste_3}{dt} = k_{25}[R_3L_1L_3\bullet\mathrm{RNaseH}] - k_{26}[L_1L_3Waste_3] + k_{-26}[L_1][L_3][Waste_3]$$

$$\frac{dWaste_3}{dt} = k_{26}[L_1L_3Waste_3] - k_{-26}[L_1][L_3][Waste_3]$$

Figure S16. Computational simulation of the inhibitor I_{M2}-gated transcription machinery-guided transient operation of two DNAzymes (state Z) shown in Figure 2. The kinetic scheme of the sub-reactions associated with the time-dependent concentration changes during the dissipative transitions is summarized in the above equations. Knowing the time-dependent concentration changes of M_1/M_2 and M_3/M_4 during the dissipative transitions, we computationally simulated the time-dependent concentration changes by using Matlab R2019b. Initial concentrations of the condition: N_3/T_3 = 0.2 μM, M_1/L_1 = 0.5 μM, M_2 = 0.5 μM, M_3/L_3 = 0.5 μM, M_4 = 0.5 μM, I_{M2} = 2 μM, NTPs = 1 mM, T7 RNAP = 0.048 μM, and RNase H = 0.266 nM. The derived rate constants are summarized in Table S6.

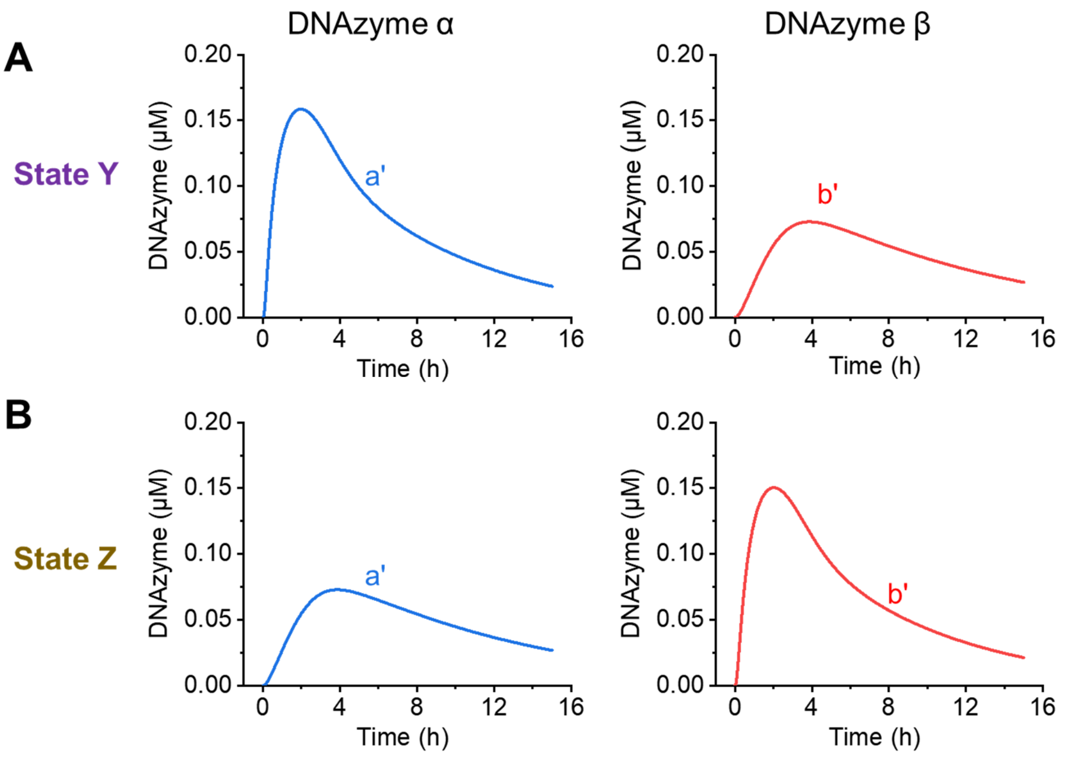

Figure S17. Computational simulation predicting the inhibitor gating efficiency for the gated dissipative DNAzyme system by using the set of reaction rates derived for Figure 3 at the different concentration of inhibitors: (A) State Y, I_{M2} = 0 μM, I_{M4} = 1 μM; (B) State Z, I_{M2} = 1 μM, I_{M4} = 0 μM. For all the system, curves a' and b' correspond to computationally-simulated temporal concentrations of DNAzyme α and DNAzyme β, respectively.

Kinetic equations of the cascaded transient operation of two DNAzymes guided by two interconnected dynamic transcription machineries in Figure 4:

(31) $N_4 + T_4 \underset{k_{-31}}{\overset{k_{31}}{\rightleftarrows}} N_4T_4$

(32) $P_1 + Q_1 \underset{k_{-32}}{\overset{k_{32}}{\rightleftarrows}} P_1Q_1$

(19) $M_3 + L_3 \underset{k_{-19}}{\overset{k_{19}}{\rightleftarrows}} M_3L_3$

(33) $N_4T_4 + \text{RNAP} \underset{k_{-33}}{\overset{k_{33}}{\rightleftarrows}} N_4T_4{\bullet}\text{RNAP}$

(34) $N_4T_4{\bullet}\text{RNAP} + \text{NTPs} \xrightarrow{k_{34}} \text{RNAP} + N_4T_4 + R_4$ (length = 66, $N_{\max}$ (U) = 23)

(35) $R_4 + M_3L_3 \underset{k_{-35}}{\overset{k_{35}}{\rightleftarrows}} R_4L_3 + M_3$

(36) $R_4L_3 + \text{RNaseH} \underset{k_{-36}}{\overset{k_{36}}{\rightleftarrows}} R_4L_3{\bullet}\text{RNaseH}$

(37) $R_4L_3{\bullet}\text{RNaseH} \xrightarrow{k_{37}} \text{RNaseH} + L_3Waste_{4-1}R_{4-2}$

(38) $L_3Waste_{4-1}R_{4-2} \underset{k_{-38}}{\overset{k_{38}}{\rightleftarrows}} L_3 + Waste_{4-1} + R_{4-2}$

(39) $R_{4-2} + P_1Q_1 \underset{k_{-39}}{\overset{k_{39}}{\rightleftarrows}} Q_1R_{4-2} + P_1$

(40) $Q_1R_{4-2} + \text{RNaseH} \underset{k_{-40}}{\overset{k_{40}}{\rightleftarrows}} Q_1R_{4-2}{\bullet}\text{RNaseH}$

(41) $Q_1R_{4-2}{\bullet}\text{RNaseH} \xrightarrow{k_{41}} \text{RNaseH} + Q_1Waste_{4-2}$

(42) $Q_1Waste_{4-2} \underset{k_{-42}}{\overset{k_{42}}{\rightleftarrows}} Q_1 + Waste_{4-2}$

(23) $M_3 + M_4 \underset{k_{-23}}{\overset{k_{23}}{\rightleftarrows}} M_3M_4$

(1) $N_1T_1 + P_1 \underset{k_{-1}}{\overset{k_1}{\rightleftarrows}} N_1T_1P_1$ (Activation)

(2) $M_1 + L_1 \underset{k_{-2}}{\overset{k_2}{\rightleftarrows}} M_1L_1$

(3) $N_1T_1P_1 + \text{RNAP} \underset{k_{-3}}{\overset{k_3}{\rightleftarrows}} N_1T_1P_1{\bullet}\text{RNAP}$

(4) $N_1T_1P_1{\bullet}\text{RNAP} + \text{NTPs} \xrightarrow{k_4} \text{RNAP} + N_1T_1P_1 + R_1$ (length = 21, $N_{\max}$ (U) = 8)

(5) $R_1 + M_1L_1 \underset{k_{-5}}{\overset{k_5}{\rightleftarrows}} R_1L_1 + M_1$

(6) $M_1 + M_2 \underset{k_{-6}}{\overset{k_6}{\rightleftarrows}} M_1M_2$

(7) $R_1L_1 + \text{RNaseH} \underset{k_{-7}}{\overset{k_7}{\rightleftarrows}} R_1L_1{\bullet}\text{RNaseH}$

(8) $R_1L_1{\bullet}\text{RNaseH} \xrightarrow{k_8} \text{RNaseH} + L_1Waste_1$

(9) $L_1Waste_1 \underset{k_{-9}}{\overset{k_9}{\rightleftarrows}} L_1 + Waste_1$

Derivatives:

$$\frac{dN_4}{dt} = k_{-31}[N_4T_4] - k_{31}[N_4][T_4]$$

$$\frac{dT_4}{dt} = k_{-31}[N_4T_4] - k_{31}[N_4][T_4]$$

$$\frac{dN_4T_4}{dt} = k_{31}[N_4][T_4] - k_{-31}[N_4T_4] - k_{33}[N_4T_4][\text{RNAP}] + k_{-33}[N_4T_4{\bullet}\text{RNAP}]$$
$$+ k_{34}[N_4T_4{\bullet}\text{RNAP}][\text{NTPs}] \times 0.07/23$$

$$\frac{dP_1}{dt} = k_{-32}[P_1Q_1] - k_{32}[P_1][Q_1] + k_{39}[R_{4-2}][P_1Q_1] - k_{-39}[Q_1R_{4-2}][P_1] - k_1[N_1T_1][P_1] + k_{-1}[N_1T_1P_1]$$

$$\frac{dQ_1}{dt} = k_{-32}[P_1Q_1] - k_{32}[P_1][Q_1] + k_{42}[Q_1Waste_{4-2}] - k_{-42}[Q_1][Waste_{4-2}]$$

$$\frac{dP_1Q_1}{dt} = k_{32}[P_1][Q_1] - k_{-32}[P_1Q_1] - k_{39}[R_{4-2}][P_1Q_1] + k_{-39}[Q_1R_{4-2}][P_1]$$

$$\frac{dM_3}{dt} = k_{-19}[M_3L_3] - k_{19}[M_3][L_3] + k_{35}[R_4][M_3L_3] - k_{-35}[R_4L_3][M_3] - k_{23}[M_3][M_4] + k_{-23}[M_3M_4]$$

$$\frac{dL_3}{dt} = k_{-19}[M_3L_3] - k_{19}[M_3][L_3] + k_{38}[L_3Waste_{4-1}R_{4-2}] - k_{-38}[L_3][Waste_{4-1}][R_{4-2}]$$

$$\frac{dM_3L_3}{dt} = k_{19}[M_3][L_3] - k_{-19}[M_3L_3] - k_{35}[R_4][M_3L_3] + k_{-35}[R_4L_3][M_3]$$

$$\frac{d\text{RNAP}}{dt} = k_{-33}[N_4T_4{\bullet}\text{RNAP}] - k_{33}[N_4T_4][\text{RNAP}] + k_{34}[N_4T_4{\bullet}\text{RNAP}][\text{NTPs}] \times 0.07/23$$
$$+ k_{-3}[N_1T_1P_1{\bullet}\text{RNAP}] - k_3[N_1T_1P_1][\text{RNAP}] + k_4[N_1T_1P_1{\bullet}\text{RNAP}][\text{NTPs}] \times 0.07/8$$

$$\frac{dN_4T_4{\bullet}\text{RNAP}}{dt} = k_{33}[N_4T_4][\text{RNAP}] - k_{-33}[N_4T_4{\bullet}\text{RNAP}] - k_{34}[N_4T_4{\bullet}\text{RNAP}][\text{NTPs}] \times 0.07/23$$

$$\frac{d\text{NTPs}}{dt} = -k_{34}[N_4T_4{\bullet}\text{RNAP}][\text{NTPs}] - k_4[N_1T_1P_1{\bullet}\text{RNAP}][\text{NTPs}]$$

$$\frac{dR_4}{dt} = k_{34}[N_4T_4{\bullet}\text{RNAP}][\text{NTPs}] \times 0.07/23 - k_{35}[R_4][M_3L_3] + k_{-35}[R_4L_3][M_3]$$

$$\frac{dR_4L_3}{dt} = k_{35}[R_4][M_3L_3] - k_{-35}[R_4L_3][M_3] - k_{36}[R_4L_3][\text{RNaseH}] + k_{-36}[R_4L_3{\bullet}\text{RNaseH}]$$

$$\frac{d\text{RNaseH}}{dt} = k_{-36}[R_4L_3{\bullet}\text{RNaseH}] - k_{36}[R_4L_3][\text{RNaseH}] + k_{37}[R_4L_3{\bullet}\text{RNaseH}] + k_{-40}[Q_1R_{4-2}{\bullet}\text{RNaseH}]$$
$$- k_{40}[Q_1R_{4-2}][\text{RNaseH}] + k_{41}[Q_1R_{4-2}{\bullet}\text{RNaseH}] + k_{-7}[R_1L_1{\bullet}\text{RNaseH}] - k_7[R_1L_1][\text{RNaseH}]$$
$$+ k_8[R_1L_1{\bullet}\text{RNaseH}]$$

$$\frac{dR_4L_3{\bullet}\text{RNaseH}}{dt} = k_{36}[R_4L_3][\text{RNaseH}] - k_{-36}[R_4L_3{\bullet}\text{RNaseH}] - k_{37}[R_4L_3{\bullet}\text{RNaseH}]$$

$$\frac{dL_3Waste_{4-1}R_{4-2}}{dt} = k_{37}[R_4L_3{\bullet}\text{RNaseH}] - k_{38}[L_3Waste_{4-1}R_{4-2}] + k_{-38}[L_3][Waste_{4-1}][R_{4-2}]$$

$$\frac{dWaste_{4-1}}{dt} = k_{38}[L_3Waste_{4-1}R_{4-2}] - k_{-38}[L_3][Waste_{4-1}][R_{4-2}]$$

$$\frac{dR_{4-2}}{dt} = k_{38}[L_3Waste_{4-1}R_{4-2}] - k_{-38}[L_3][Waste_{4-1}][R_{4-2}] - k_{39}[R_{4-2}][P_1Q_1] + k_{-39}[Q_1R_{4-2}][P_1]$$

$$\frac{dQ_1R_{4-2}}{dt} = k_{39}[R_{4-2}][P_1Q_1] - k_{-39}[Q_1R_{4-2}][P_1] - k_{40}[Q_1R_{4-2}][\text{RNaseH}] + k_{-40}[Q_1R_{4-2}\bullet\text{RNaseH}]$$

$$\frac{dQ_1R_{4-2}\bullet\text{RNaseH}}{dt} = k_{40}[Q_1R_{4-2}][\text{RNaseH}] - k_{-40}[Q_1R_{4-2}\bullet\text{RNaseH}] - k_{41}[Q_1R_{4-2}\bullet\text{RNaseH}]$$

$$\frac{dQ_1Waste_{4-2}}{dt} = k_{41}[Q_1R_{4-2}\bullet\text{RNaseH}] - k_{42}[Q_1Waste_{4-2}] + k_{-42}[Q_1][Waste_{4-2}]$$

$$\frac{dWaste_{4-2}}{dt} = k_{42}[Q_1Waste_{4-2}] - k_{-42}[Q_1][Waste_{4-2}]$$

$$\frac{dM_4}{dt} = k_{-23}[M_3M_4] - k_{23}[M_3][M_4]$$

$$\frac{dM_3M_4}{dt} = k_{23}[M_3][M_4] - k_{-23}[M_3M_4]$$

$$\frac{dN_1T_1}{dt} = k_{-1}[N_1T_1P_1] - k_1[N_1T_1][P_1]$$

$$\frac{dN_1T_1P_1}{dt} = k_1[N_1T_1][P_1] - k_{-1}[N_1T_1P_1] - k_3[N_1T_1P_1][\text{RNAP}] + k_{-3}[N_1T_1P_1\bullet\text{RNAP}] + k_4[N_1T_1P_1\bullet\text{RNAP}][\text{NTPs}]\times 0.07/8$$

$$\frac{dM_1}{dt} = k_{-2}[M_1L_1] - k_2[M_1][L_1] + k_{-6}[M_1M_2] - k_6[M_1][M_2] + k_5[R_1][M_1L_1] - k_{-5}[R_1L_1][M_1]$$

$$\frac{dL_1}{dt} = k_{-2}[M_1L_1] - k_2[M_1][L_1] + k_9[L_1Waste_1] - k_{-9}[L_1][Waste_1]$$

$$\frac{dM_1L_1}{dt} = k_2[M_1][L_1] - k_{-2}[M_1L_1] - k_5[R_1][M_1L_1] + k_{-5}[R_1L_1][M_1]$$

$$\frac{dN_1T_1P_1\bullet\text{RNAP}}{dt} = k_3[N_1T_1P_1][\text{RNAP}] - k_{-3}[N_1T_1P_1\bullet\text{RNAP}] - k_4[N_1T_1P_1\bullet\text{RNAP}][\text{NTPs}]\times 0.07/8$$

$$\frac{dR_1}{dt} = k_4[N_1T_1P_1\bullet\text{RNAP}][\text{NTPs}]\times 0.07/8 - k_5[R_1][M_1L_1] + k_{-5}[R_1L_1][M_1]$$

$$\frac{dR_1L_1}{dt} = k_5[R_1][M_1L_1] - k_{-5}[R_1L_1][M_1] - k_7[R_1L_1][\text{RNaseH}] + k_{-7}[R_1L_1\bullet\text{RNaseH}]$$

$$\frac{dM_2}{dt} = k_{-6}[M_1M_2] - k_6[M_1][M_2]$$

$$\frac{dM_1M_2}{dt} = k_6[M_1][M_2] - k_{-6}[M_1M_2]$$

$$\frac{dR_1L_1\bullet\text{RNaseH}}{dt} = k_7[R_1L_1][\text{RNaseH}] - k_{-7}[R_1L_1\bullet\text{RNaseH}] - k_8[R_1L_1\bullet\text{RNaseH}]$$

$$\frac{dL_1Waste_1}{dt} = k_8[R_1L_1\bullet\text{RNaseH}] - k_9[L_1Waste_1] + k_{-9}[L_1][Waste_1]$$

$$\frac{dWaste_1}{dt} = k_9[L_1Waste_1] - k_{-9}[L_1][Waste_1]$$

Figure S18. Computational simulation of the transcription machinery-guided, transient, cascaded operation of dissipative DNAzymes in Figure 4. The kinetic scheme of the sub-reactions associated with the time-dependent concentration changes during the

dissipative transitions is summarized in the above equations. Knowing the time-dependent concentration changes of M_1/M_2 and M_3/M_4, during the dissipative transitions, we computationally simulated the time-dependent concentration changes by using Matlab R2019b. Initial concentrations of the condition: N_4/T_4 = 0.2 μM, N_1/T_1= 0.2 μM, M_1/L_1 = 0.5 μM, M_2 = 0.5 μM, M_3/L_3 = 0.5 μM, M_4 = 0.5 μM, P_1/Q_1 = 0.5 μM, NTPs = 1 mM, T7 RNAP = 0.048 μM, and RNase H = 0.332 nM. The derived rate constants are summarized in Table S7.

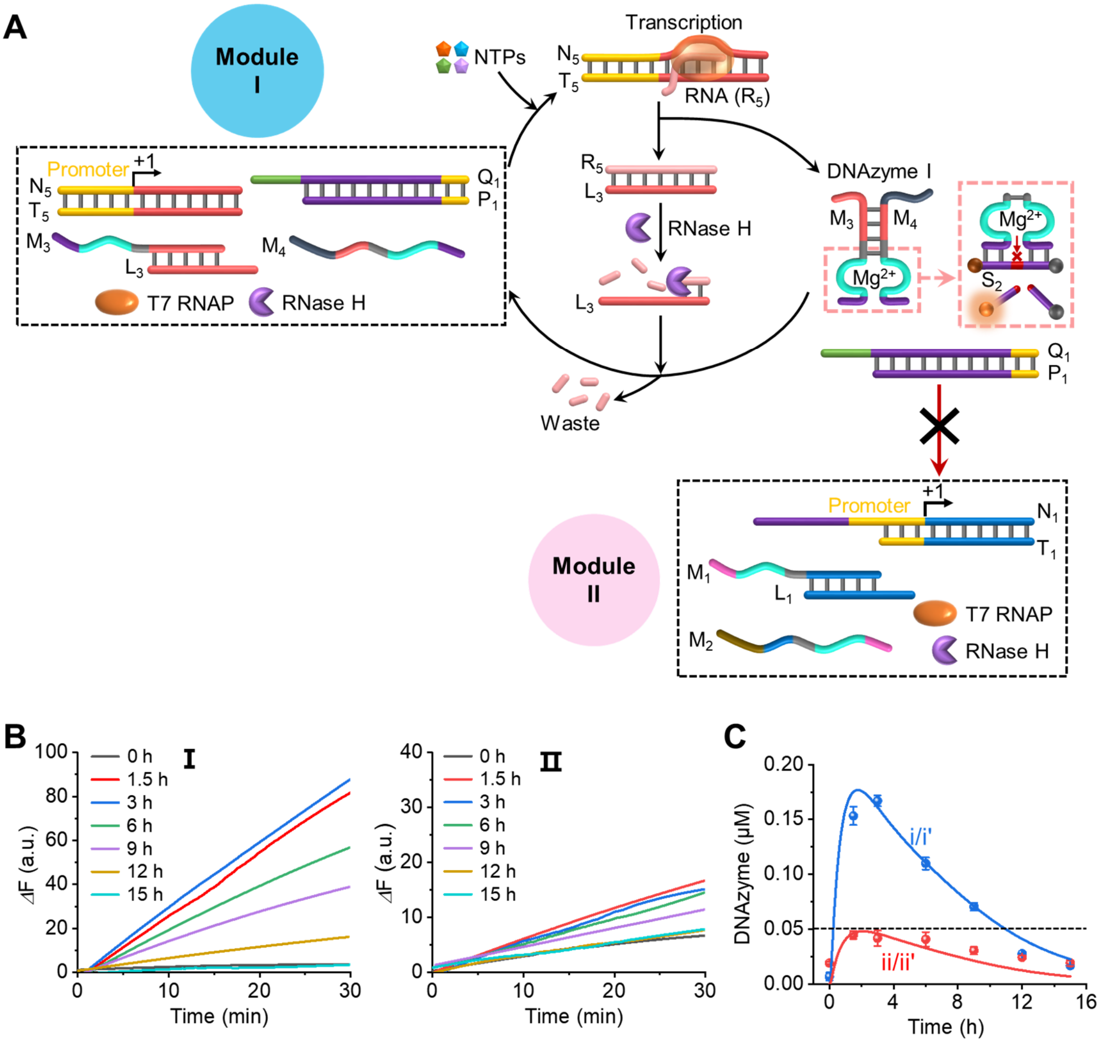

Figure S19. (A) Schematic illustration showing a control study involving a modified template (N_5/T_5) in module I that lacks the key sequence to operate the two-layer cascade which is driven by interconnected dynamic transcription machineries. (B) Panel I: Time-dependent fluorescence changes generated from the cleavage of S_2 by DNAzyme β formed at time-intervals of the transient operation of the control reaction module. Panel II: Time-dependent fluorescence changes generated from the cleavage of S_1 by DNAzyme α formed at time-intervals of the transient operation of the control reaction module. (C) Dotted curves i and ii, transient concentrations of DNAzyme β and DNAzyme α, respectively; solid curves i′ and ii', computationally fitted transient concentrations of the DNAzyme β and DNAzyme α, respectively, by using the kinetic model in Figure S20. The experimental conditions are: $N_5/T_5 = 0.2$ μM, $N_1/T_1 = 0.2$ μM, $Q_1/P_1 = 0.5$ μM, $M_1/L_1 = 0.5$ μM, $M_3/L_3 = 0.5$ μM, $M_2 = 0.5$ μM, $M_4 = 0.5$ μM, NTPs = 1 mM, T7 RNAP = 3 U/μL (0.048 μM), RNase H = 10 U/mL (0.332 nM).

A control experiment emphasizing the interrelation between module I and module II to operate the two-layer cascade

The operation of the two-layer cascade driven by interconnected dynamic transcription machineries shown in Figure 4, employed the transcription template N_4/T_4, in module I, that was engineered to yield, upon transcription, the product R_4 which is responsible to displace L_3 (from L_3/M_3) and Q_1 (from Q_1/P_1). While the displacement of L_3 results in the transient operation of module I, the displacement of Q_1 yields P_1 that intercommunicates the two modules. Accordingly, for the control experiment in Figure S19, a modified transcription template N_5/T_5 was designed. This transcription template includes the duplex sequence that activates module I, yet it lacks the domain to transcribe, in the RNA product R_5, the sequence to separate Q_1/P_1. Thus, in the presence of this transcription system, the formation of the inter-connecting strand P_1 is prohibited, and therefore only DNAzyme β is formed. Indeed, Figure S19C demonstrates that the control system, composed of module I (containing the modified template N_5/T_5) and module II, yields predominantly DNAzyme β and only traces of DNAzyme α (generated by the inefficient self-operation of template N_1/T_1).

Kinetic equations of the control study shown in Figure S19:

(43) $N_5 + T_5 \underset{k_{-43}}{\overset{k_{43}}{\rightleftarrows}} N_5T_5$

(32) $P_1 + Q_1 \underset{k_{-32}}{\overset{k_{32}}{\rightleftarrows}} P_1Q_1$

(19) $M_3 + L_3 \underset{k_{-19}}{\overset{k_{19}}{\rightleftarrows}} M_3L_3$

(44) N_5T_5 + RNAP $\underset{k_{-44}}{\overset{k_{44}}{\rightleftarrows}} N_5T_5$•RNAP

(45) N_5T_5•RNAP + NTPs $\xrightarrow{k_{45}}$ RNAP + $N_5T_5 + R_5$ (length = 23, N_{max} (G) = 10)

(46) $R_5 + M_3L_3 \underset{k_{-46}}{\overset{k_{46}}{\rightleftarrows}} R_5L_3 + M_3$

(23) $M_3 + M_4 \underset{k_{-23}}{\overset{k_{23}}{\rightleftarrows}} M_3M_4$

(47) R_5L_3 + RNaseH $\underset{k_{-47}}{\overset{k_{47}}{\rightleftarrows}} R_5L_3$•RNaseH

(48) R_5L_3•RNaseH $\xrightarrow{k_{48}}$ RNaseH + L_3Waste_5

(49) $L_3Waste_5 \underset{k_{-49}}{\overset{k_{49}}{\rightleftarrows}} L_3 + Waste_5$

(50) $N_1T_1 + P_1Q_1 \underset{k_{-50}}{\overset{k_{50}}{\rightleftarrows}} N_1T_1P_1 + Q_1$

(2) $M_1 + L_1 \underset{k_{-2}}{\overset{k_{2}}{\rightleftarrows}} M_1L_1$

(3) $N_1T_1P_1$ + RNAP $\underset{k_{-3}}{\overset{k_{3}}{\rightleftarrows}} N_1T_1P_1$•RNAP (fast)

(4) $N_1T_1P_1$•RNAP + NTPs $\xrightarrow{k_{4}}$ RNAP + $N_1T_1P_1 + R_1$ (fast)

(51) N_1T_1 + RNAP $\underset{k_{-51}}{\overset{k_{51}}{\rightleftarrows}} N_1T_1$•RNAP (slow)

(52) N_1T_1•RNAP + NTPs $\xrightarrow{k_{52}}$ RNAP + $N_1T_1 + R_1$ (slow)

(5) $R_1 + M_1L_1 \underset{k_{-5}}{\overset{k_{5}}{\rightleftarrows}} R_1L_1 + M_1$

(6) $M_1 + M_2 \underset{k_{-6}}{\overset{k_{6}}{\rightleftarrows}} M_1M_2$

(7) R_1L_1 + RNaseH $\underset{k_{-7}}{\overset{k_{7}}{\rightleftarrows}} R_1L_1$•RNaseH

(8) R_1L_1•RNaseH $\xrightarrow{k_{8}}$ RNaseH + L_1Waste_1

(9) $L_1Waste_1 \underset{k_{-9}}{\overset{k_{9}}{\rightleftarrows}} L_1 + Waste_1$

Derivatives:

$$\frac{dN_5}{dt} = k_{-43}[N_5T_5] - k_{43}[N_5][T_5]$$

$$\frac{dT_5}{dt} = k_{-43}[N_5T_5] - k_{43}[N_5][T_5]$$

$$\frac{dN_5T_5}{dt} = k_{43}[N_5][T_5] - k_{-43}[N_5T_5] - k_{44}[N_5T_5][\mathrm{RNAP}] + k_{-44}[N_5T_5\bullet\mathrm{RNAP}]$$
$$+ k_{45}[N_5T_5\bullet\mathrm{RNAP}][\mathrm{NTPs}] \times 0.07/10$$

$$\frac{dP_1}{dt} = k_{-32}[P_1Q_1] - k_{32}[P_1][Q_1]$$

$$\frac{dQ_1}{dt} = k_{-32}[P_1Q_1] - k_{32}[P_1][Q_1] + k_{50}[N_1T_1][P_1Q_1] - k_{-50}[N_1T_1P_1][Q_1]$$

$$\frac{dP_1Q_1}{dt} = k_{32}[P_1][Q_1] - k_{-32}[P_1Q_1] - k_{50}[N_1T_1][P_1Q_1] + k_{-50}[N_1T_1P_1][Q_1]$$

$$\frac{dM_3}{dt} = k_{-19}[M_3L_3] - k_{19}[M_3][L_3] + k_{46}[R_5][M_3L_3] - k_{-46}[R_5L_3][M_3] - k_{23}[M_3][M_4] + k_{-23}[M_3M_4]$$

$$\frac{dL_3}{dt} = k_{-19}[M_3L_3] - k_{19}[M_3][L_3] + k_{49}[L_3Waste_5] - k_{-49}[L_3][Waste_5]$$

$$\frac{dM_3L_3}{dt} = k_{19}[M_3][L_3] - k_{-19}[M_3L_3] - k_{46}[R_5][M_3L_3] + k_{-46}[R_5L_3][M_3]$$

$$\frac{d\text{RNAP}}{dt} = k_{-44}[N_5T_5\bullet\text{RNAP}] - k_{44}[N_5T_5][\text{RNAP}] + k_{45}[N_5T_5\bullet\text{RNAP}][\text{NTPs}]\times 0.07/10$$
$$- k_3[N_1T_1P_1][\text{RNAP}] + k_{-3}[N_1T_1P_1\bullet\text{RNAP}] + k_4[N_1T_1P_1\bullet\text{RNAP}][\text{NTPs}]\times 0.07/8$$
$$+ k_{-51}[N_1T_1\bullet\text{RNAP}] - k_{51}[N_1T_1][\text{RNAP}] + k_{52}[N_1T_1\bullet\text{RNAP}][\text{NTPs}]\times 0.07/8$$

$$\frac{dN_5T_5\bullet\text{RNAP}}{dt} = k_{44}[N_5T_5][\text{RNAP}] - k_{-44}[N_5T_5\bullet\text{RNAP}] - k_{45}[N_5T_5\bullet\text{RNAP}][\text{NTPs}]\times 0.07/10$$

$$\frac{d\text{NTPs}}{dt} = -k_{45}[N_5T_5\bullet\text{RNAP}][\text{NTPs}] - k_4[N_1T_1P_1\bullet\text{RNAP}][\text{NTPs}] - k_{52}[N_1T_1\bullet\text{RNAP}][\text{NTPs}]$$

$$\frac{dR_5}{dt} = k_{45}[N_5T_5\bullet\text{RNAP}][\text{NTPs}]\times 0.07/10 - k_{46}[R_5][M_3L_3] + k_{-46}[R_5L_3][M_3]$$

$$\frac{dR_5L_3}{dt} = k_{46}[R_5][M_3L_3] - k_{-46}[R_5L_3][M_3] - k_{47}[R_5L_3][\text{RNaseH}] + k_{-47}[R_5L_3\bullet\text{RNaseH}]$$

$$\frac{dM_4}{dt} = k_{-23}[M_3M_4] - k_{23}[M_3][M_4]$$

$$\frac{dM_3M_4}{dt} = k_{23}[M_3][M_4] - k_{-23}[M_3M_4]$$

$$\frac{d\text{RNaseH}}{dt} = k_{-47}[R_5L_3\bullet\text{RNaseH}] - k_{47}[R_5L_3][\text{RNaseH}] + k_{48}[R_5L_3\bullet\text{RNaseH}]$$
$$+ k_{-7}[R_1L_1\bullet\text{RNaseH}] - k_7[R_1L_1][\text{RNaseH}] + k_8[R_1L_1\bullet\text{RNaseH}]$$

$$\frac{dR_5L_3\bullet\text{RNaseH}}{dt} = k_{47}[R_5L_3][\text{RNaseH}] - k_{-47}[R_5L_3\bullet\text{RNaseH}] - k_{48}[R_5L_3\bullet\text{RNaseH}]$$

$$\frac{dL_3Waste_5}{dt} = k_{48}[R_5L_3\bullet\text{RNaseH}] - k_{49}[L_3Waste_5] + k_{-49}[L_3][Waste_5]$$

$$\frac{dWaste_5}{dt} = k_{49}[L_3Waste_5] - k_{-49}[L_3][Waste_5]$$

$$\frac{dN_1T_1}{dt} = k_{-50}[N_1T_1P_1][Q_1] - k_{50}[N_1T_1][P_1Q_1] - k_{51}[N_1T_1][\text{RNAP}] + k_{-51}[N_1T_1\bullet\text{RNAP}]$$
$$+ k_{52}[N_1T_1\bullet\text{RNAP}][\text{NTPs}]\times 0.07/8$$

$$\frac{dN_1T_1P_1}{dt} = k_{50}[N_1T_1][P_1Q_1] - k_{-50}[N_1T_1P_1][Q_1] - k_3[N_1T_1P_1][\text{RNAP}] + k_{-3}[N_1T_1P_1\bullet\text{RNAP}]$$
$$+ k_4[N_1T_1P_1\bullet\text{RNAP}][\text{NTPs}]\times 0.07/8$$

$$\frac{dM_1}{dt} = k_{-2}[M_1L_1] - k_2[M_1][L_1] + k_{-6}[M_1M_2] - k_6[M_1][M_2] + k_5[R_1][M_1L_1] - k_{-5}[R_1L_1][M_1]$$

$$\frac{dL_1}{dt} = k_{-2}[M_1L_1] - k_2[M_1][L_1] + k_9[L_1Waste_1] - k_{-9}[L_1][Waste_1]$$

$$\frac{dM_1L_1}{dt} = k_2[M_1][L_1] - k_{-2}[M_1L_1] - k_5[R_1][M_1L_1] + k_{-5}[R_1L_1][M_1]$$

$$\frac{dN_1T_1P_1\bullet\text{RNAP}}{dt} = k_3[N_1T_1P_1][\text{RNAP}] - k_{-3}[N_1T_1P_1\bullet\text{RNAP}] - k_4[N_1T_1P_1\bullet\text{RNAP}][\text{NTPs}]\times 0.07/8$$

$$\frac{dN_1T_1\bullet\text{RNAP}}{dt} = k_{51}[N_1T_1][\text{RNAP}] - k_{-51}[N_1T_1\bullet\text{RNAP}] - k_{52}[N_1T_1\bullet\text{RNAP}][\text{NTPs}]\times 0.07/8$$

$$\frac{dR_1}{dt} = k_4[N_1T_1P_1\bullet\text{RNAP}][\text{NTPs}]\times 0.07/8 + k_{52}[N_1T_1\bullet\text{RNAP}][\text{NTPs}]\times 0.07/8 - k_5[R_1][M_1L_1]$$
$$+ k_{-5}[R_1L_1][M_1]$$

$$\frac{dR_1L_1}{dt} = k_5[R_1][M_1L_1] - k_{-5}[R_1L_1][M_1] - k_7[R_1L_1][\text{RNaseH}] + k_{-7}[R_1L_1\bullet\text{RNaseH}]$$

$$\frac{dM_2}{dt} = k_{-6}[M_1M_2] - k_6[M_1][M_2]$$

$$\frac{dM_1M_2}{dt} = k_6[M_1][M_2] - k_{-6}[M_1M_2]$$

$$\frac{dR_1L_1\bullet\text{RNaseH}}{dt} = k_7[R_1L_1][\text{RNaseH}] - k_{-7}[R_1L_1\bullet\text{RNaseH}] - k_8[R_1L_1\bullet\text{RNaseH}]$$

$$\frac{dL_1Waste_1}{dt} = k_8[R_1L_1\bullet\text{RNaseH}] - k_9[L_1Waste_1] + k_{-9}[L_1][Waste_1]$$

$$\frac{dWaste_1}{dt} = k_9[L_1Waste_1] - k_{-9}[L_1][Waste_1]$$

Figure S20. Computational simulation of the control study shown in Figure S19. The kinetic scheme of the sub-reactions associated with the time-dependent concentration changes during the dissipative transitions is summarized in the above equations. Knowing the time-dependent concentration changes of M_1/M_2 and M_3/M_4, we computationally simulated the time-dependent concentration changes by using Matlab R2019b. Initial concentrations of the condition: N_5/T_5 = 0.2 μM, N_1/T_1 = 0.2 μM, Q_1/P_1 = 0.5 μM, M_1/L_1 = 0.5 μM, M_3/L_3 = 0.5 μM, M_2 = 0.5 μM, M_4 = 0.5 μM, NTPs = 1 mM, T7 RNAP = 0.048 μM, RNase H = 0.332 nM. The derived rate constants are summarized in Table S8.

3. Supplemental Tables

Table S1. Rate constants derived from the computational simulation of the transcription machinery-guided transient operation of a DNAzyme shown in Figure 1 (T = 33 °C).

k_1	20.2 $\mu M^{-1} min^{-1}$	k_4	0.3 $\mu M^{-1} min^{-1}$	k_{-7}	1.0×10^{-6} min^{-1}
k_{-1}	2.0×10^{-2} min^{-1}	k_5	10.0 $\mu M^{-1} min^{-1}$	k_8	90.0 min^{-1}
k_2	18.6 $\mu M^{-1} min^{-1}$	k_{-5}	5.3 $\mu M^{-1} min^{-1}$	k_9	2.0 min^{-1}
k_{-2}	1.0×10^{-6} min^{-1}	k_6	1.5×10^{-1} $\mu M^{-1} min^{-1}$	k_{-9}	1.0×10^{-2} $\mu M^{-1} min^{-1}$
k_3	2.8 μM^{-1} min^{-1}	k_{-6}	1.0×10^{-2} min^{-1}		
k_{-3}	1.0×10^{-6} min^{-1}	k_7	1.0×10^{3} $\mu M^{-1} min^{-1}$		

Table S2. Rate constants derived from the computational simulation of real-time fluorescence monitoring of the transcription shown in Figure S7 (T = 33 °C).

k_{10}	20.2 $\mu M^{-1} min^{-1}$	k_{-11}	1.0×10^{-6} min^{-1}	k_{13}	2.4×10^{-1} $\mu M^{-1} min^{-1}$
k_{-10}	2.0×10^{-2} min^{-1}	k_{12}	2.8 μM^{-1} min^{-1}	k_{14}	10.0 $\mu M^{-1} min^{-1}$
k_{11}	18.6 $\mu M^{-1} min^{-1}$	k_{-12}	1.0×10^{-6} min^{-1}	k_{-14}	5.3 $\mu M^{-1} min^{-1}$

Experimentally obtained values: k_{11} = 18.5 μM^{-1} min^{-1}, k_{-11} = 1.0×10^{-6} min^{-1}.

Table S3. Rate constants derived from the computational simulation of transcription machinery-guided dissipative synthesis and degradation of RNA shown in Figure S9 (T = 33 °C).

k_{10}	20.2 $\mu M^{-1} min^{-1}$	k_{-12}	1.0×10^{-6} min^{-1}	k_{-15}	1.0×10^{-6} min^{-1}
k_{-10}	2.0×10^{-2} min^{-1}	k_{13}	2.4×10^{-1} $\mu M^{-1} min^{-1}$	k_{16}	55 min^{-1}
k_{11}	18.6 $\mu M^{-1} min^{-1}$	k_{14}	10.0 $\mu M^{-1} min^{-1}$	k_{17}	2.0 min^{-1}
k_{-11}	1.0×10^{-6} min^{-1}	k_{-14}	5.3 $\mu M^{-1} min^{-1}$	k_{-17}	1.0×10^{-2} $\mu M^{-1} min^{-1}$
k_{12}	2.8 μM^{-1} min^{-1}	k_{15}	1.0×10^{3} $\mu M^{-1} min^{-1}$		

Experimentally obtained values: k_{11} = 18.5 μM^{-1} min^{-1}, k_{-11} = 1.0×10^{-6} min^{-1}.

Table S4. Rate constants derived from the computational simulation of the transcription machinery-guided, parallel, non-gated transient operation of two DNAzymes (state X) shown in Figure 2 (T = 33 °C).

k_{18}	52.9 $\mu M^{-1} min^{-1}$	k_{-20}	1.0×10^{-6} min^{-1}	k_{-23}	1.0 $\times10^{-2}$ min^{-1}
k_{-18}	1.0×10^{-6} min^{-1}	k_{21}	0.1 $\mu M^{-1} min^{-1}$	k_{24}	1.0×10^{3} $\mu M^{-1} min^{-1}$
k_{2}	18.6 $\mu M^{-1} min^{-1}$	k_{22}	10.0 $\mu M^{-2} min^{-1}$	k_{-24}	1.0×10^{-6} min^{-1}
k_{-2}	1.0×10^{-6} min^{-1}	k_{-22}	5.0 $\mu M^{-2} min^{-1}$	k_{25}	53.0 min^{-1}
k_{19}	18.6 $\mu M^{-1} min^{-1}$	k_{6}	1.5×10^{-1} $\mu M^{-1} min^{-1}$	k_{26}	0.5 min^{-1}
k_{-19}	1.0×10^{-6} min^{-1}	k_{-6}	1.0×10^{-2} min^{-1}	k_{-26}	0.3 $\mu M^{-2} min^{-1}$
k_{20}	2.8 μM^{-1} min^{-1}	k_{23}	1.2×10^{-1} $\mu M^{-1} min^{-1}$		

Table S5. Rate constants derived from the computational simulation of the transcription machinery-guided, inhibitor I_{M4}-gated transient operation of two DNAzymes (state Y) shown in Figure 2 (T = 33 °C).

k_{18}	52.9 $\mu M^{-1} min^{-1}$	k_{21}	0.1 $\mu M^{-1} min^{-1}$	k_{-28}	4.0×10^{-3} $\mu M^{-1} min^{-1}$
k_{-18}	1.0×10^{-6} min^{-1}	k_{22}	10.0 $\mu M^{-2} min^{-1}$	k_{24}	1.0×10^{3} $\mu M^{-1} min^{-1}$
k_{2}	18.6 $\mu M^{-1} min^{-1}$	k_{-22}	5.0 $\mu M^{-2} min^{-1}$	k_{-24}	1.0×10^{-6} min^{-1}
k_{-2}	1.0×10^{-6} min^{-1}	k_{6}	1.5×10^{-1} $\mu M^{-1} min^{-1}$	k_{25}	53.0 min^{-1}
k_{19}	18.6 $\mu M^{-1} min^{-1}$	k_{-6}	1.0 $\times10^{-2}$ min^{-1}	k_{26}	0.5 min^{-1}
k_{-19}	1.0×10^{-6} min^{-1}	k_{27}	20.0 $\mu M^{-1} min^{-1}$	k_{-26}	0.3 $\mu M^{-2} min^{-1}$
k_{20}	2.8 μM^{-1} min^{-1}	k_{-27}	1.0×10^{-6} min^{-1}		
k_{-20}	1.0×10^{-6} min^{-1}	k_{28}	9.0×10^{-3} $\mu M^{-1} min^{-1}$		

Table S6. Rate constants derived from the computational simulation of the transcription machinery-guided, inhibitor I_{M2}-gated transient operation of two DNAzymes (state Z) shown in Figure 2 (T = 33 °C).

k_{18}	52.9 $\mu M^{-1}min^{-1}$	k_{21}	0.1 $\mu M^{-1}min^{-1}$	k_{-23}	1.0 $\times 10^{-2}$ min^{-1}
k_{-18}	1.0×10^{-6} min^{-1}	k_{22}	10.0 $\mu M^{-2}min^{-1}$	k_{24}	1.0×10^{3} $\mu M^{-1}min^{-1}$
k_{2}	18.6 $\mu M^{-1}min^{-1}$	k_{-22}	5.0 $\mu M^{-2}min^{-1}$	k_{-24}	1.0×10^{-6} min^{-1}
k_{-2}	1.0×10^{-6} min^{-1}	k_{29}	20.0 $\mu M^{-1}min^{-1}$	k_{25}	53.0 min^{-1}
k_{19}	18.6 $\mu M^{-1}min^{-1}$	k_{-29}	1.0×10^{-6} min^{-1}	k_{26}	0.5 min^{-1}
k_{-19}	1.0×10^{-6} min^{-1}	k_{30}	9.0×10^{-3} $\mu M^{-1}min^{-1}$	k_{-26}	0.3 $\mu M^{-2}min^{-1}$
k_{20}	2.8 μM^{-1} min^{-1}	k_{-30}	4.0×10^{-3} $\mu M^{-1}min^{-1}$		
k_{-20}	1.0×10^{-6} min^{-1}	k_{23}	1.2×10^{-1} $\mu M^{-1}min^{-1}$		

Table S7. Rate constants derived from the computational simulation of the cascaded transient operation of two DNAzymes guided by two interconnected dynamic transcription machineries in Figure 4 (T = 33 °C).

k_{31}	79.5 μM^{-1} min^{-1}	k_{38}	1.2×10^{-1} min^{-1}	k_{-2}	1.0×10^{-6} min^{-1}
k_{-31}	1.0×10^{-6} min^{-1}	k_{-38}	1.0×10^{-2} $\mu M^{-2}min^{-1}$	k_{3}	2.8 μM^{-1} min^{-1}
k_{32}	46.2 μM^{-1} min^{-1}	k_{39}	10.0 $\mu M^{-1}min^{-1}$	k_{-3}	1.0×10^{-6} min^{-1}
k_{-32}	1.0×10^{-6} min^{-1}	k_{-39}	5.0 $\mu M^{-1}min^{-1}$	k_{4}	0.3 $\mu M^{-1}min^{-1}$
k_{19}	18.6 μM^{-1} min^{-1}	k_{40}	1.0×10^{3} $\mu M^{-1}min^{-1}$	k_{5}	10.0 $\mu M^{-1}min^{-1}$
k_{-19}	1.0×10^{-6} min^{-1}	k_{-40}	1.0×10^{-6} min^{-1}	k_{-5}	5.3 $\mu M^{-1}min^{-1}$
k_{33}	3.0 μM^{-1} min^{-1}	k_{41}	50.0 min^{-1}	k_{6}	1.5×10^{-1} $\mu M^{-1}min^{-1}$
k_{-33}	1.0×10^{-6} min^{-1}	k_{42}	0.6 min^{-1}	k_{-6}	1.0×10^{-2} min^{-1}
k_{34}	0.1 $\mu M^{-1}min^{-1}$	k_{-42}	0.1 $\mu M^{-1}min^{-1}$	k_{7}	1.0×10^{3} $\mu M^{-1}min^{-1}$
k_{35}	10.0 $\mu M^{-1}min^{-1}$	k_{23}	1.2×10^{-1} $\mu M^{-1}min^{-1}$	k_{-7}	1.0×10^{-6} min^{-1}
k_{-35}	5.3 $\mu M^{-1}min^{-1}$	k_{-23}	1.0×10^{-2} min^{-1}	k_{8}	90.0 min^{-1}
k_{36}	1.0×10^{3} $\mu M^{-1}min^{-1}$	k_{1}	20.2 $\mu M^{-1}min^{-1}$	k_{9}	2.0 min^{-1}
k_{-36}	1.0×10^{-6} min^{-1}	k_{-1}	2.0×10^{-2} min^{-1}	k_{-9}	1.0×10^{-2} $\mu M^{-1}min^{-1}$
k_{37}	65.0 min^{-1}	k_{2}	18.6 $\mu M^{-1}min^{-1}$		

Table S8. Rate constants derived from the computational simulation of the control study shown in Figure S19 (T = 33 °C).

k_{43}	48.3 μM^{-1} min^{-1}	k_{47}	1.0×10^{3} μM^{-1}min^{-1}	k_{-51}	1.0×10^{-6} min^{-1}
k_{-43}	1.0×10^{-6} min^{-1}	k_{-47}	1.0×10^{-6} min^{-1}	k_{52}	7.0×10^{-2} μM^{-1}min^{-1}
k_{32}	46.2 μM^{-1} min^{-1}	k_{48}	85.0 min^{-1}	k_{5}	10.0 μM^{-1}min^{-1}
k_{-32}	1.0×10^{-6} min^{-1}	k_{49}	2.5×10^{-1} min^{-1}	k_{-5}	5.3 μM^{-1}min^{-1}
k_{19}	18.6 μM^{-1}min^{-1}	k_{-49}	0.1 μM^{-1}min^{-1}	k_{6}	1.5×10^{-1} μM^{-1}min^{-1}
k_{-19}	1.0×10^{-6} min^{-1}	k_{50}	1.0×10^{-3} μM^{-1}min^{-1}	k_{-6}	1.0×10^{-2} min^{-1}
k_{44}	3.0 μM^{-1} min^{-1}	k_{-50}	2.5 μM^{-1}min^{-1}	k_{7}	1.0×10^{3} μM^{-1}min^{-1}
k_{-44}	1.0×10^{-6} min^{-1}	k_{2}	18.6 μM^{-1}min^{-1}	k_{-7}	1.0×10^{-6} min^{-1}
k_{45}	0.2 μM^{-1}min^{-1}	k_{-2}	1.0×10^{-6} min^{-1}	k_{8}	90.0 min^{-1}
k_{46}	10.0 μM^{-1}min^{-1}	k_{3}	2.8 μM^{-1} min^{-1}	k_{9}	2.0 min^{-1}
k_{-46}	5.3 μM^{-1}min^{-1}	k_{-3}	1.0×10^{-6} min^{-1}	k_{-9}	1.0×10^{-2} μM^{-1}min^{-1}
k_{23}	1.2×10^{-1} μM^{-1}min^{-1}	k_{4}	0.3 μM^{-1}min^{-1}		
k_{-23}	1.0×10^{-2} min^{-1}	k_{51}	0.6 μM^{-1}min^{-1}		

4. Supplemental References

(1) Feyrer, H.; Munteanu, R.; Baronti, L.; Petzold, K. One-Pot Production of RNA in High Yield and Purity Through Cleaving Tandem Transcripts. *Molecules* **2020**, *25*, 1142.
